# Clinical Features and Serum Biomarkers in HIV Immune Reconstitution Inflammatory Syndrome after Cryptococcal Meningitis: A Prospective Cohort Study

**DOI:** 10.1371/journal.pmed.1000384

**Published:** 2010-12-21

**Authors:** David R. Boulware, David B. Meya, Tracy L. Bergemann, Darin L. Wiesner, Joshua Rhein, Abdu Musubire, Sarah J. Lee, Andrew Kambugu, Edward N. Janoff, Paul R. Bohjanen

**Affiliations:** 1 Division of Infectious Diseases & International Medicine, Department of Medicine, University of Minnesota, Minneapolis, Minnesota, United States of America; 2 Center for Infectious Diseases and Microbiology Translational Research, University of Minnesota, Minneapolis, Minnesota, United States of America; 3 Infectious Disease Institute, Makerere University, Kampala, Uganda; 4 Division of Biostatistics, School of Public Health, University of Minnesota, Minneapolis, Minnesota, United States of America; 5 Mucosal and Vaccine Research Program Colorado (MAVRC), Division of Infectious Diseases, University of Colorado Denver School of Medicine, Denver Veterans Affairs Medical Center, Denver, Colorado, United States of America; 6 Department of Microbiology, University of Minnesota, Minneapolis, Minnesota, United States of America; Liverpool School of Tropical Medicine, United Kingdom

## Abstract

**Background:**

Although antiretroviral therapy (ART) improves survival in persons with cryptococcal meningitis (CM) and AIDS, ART frequently elicits HIV immune reconstitution inflammatory syndrome (IRIS), an exaggerated and frequently deadly inflammatory reaction that complicates recovery from immunodeficiency. The pathogenesis of IRIS is poorly understood and prediction of IRIS is not possible.

**Methods and Findings:**

We prospectively followed 101 ART-naïve Ugandans with AIDS and recent CM for one year after initiating ART, and used Luminex multiplex assays to compare serum cytokine levels in participants who did or did not develop IRIS. IRIS occurred in 45% of participants with recent CM on ART, including 30% with central nervous system (CNS) manifestations. The median time to CM-IRIS was 8.8 wk on ART. Overall mortality on ART was 36% with IRIS and 21% without IRIS. CM-IRIS was independently associated with death (HR = 2.3, 95% CI 1.1–5.1, *p* = 0.04). Patients experiencing subsequent CM-IRIS had 4-fold higher median serum cryptococcal antigen (CRAG) levels pre-ART (*p* = 0.006). Higher pre-ART levels of interleukin (IL)-4 and IL-17 as well as lower tumor necrosis factor (TNF)-α, granulocyte colony-stimulating factor (G-CSF), granulocyte-macrophage colony-stimulating factor (GM-CSF), and vascular endothelial growth factor (VEGF) predicted future IRIS in multivariate analyses (area under the curve [AUC] = 0.82). An algorithm based on seven pre-ART serum biomarkers was a robust tool for stratifying high (83%), moderate (48%), and low risk (23%) for IRIS in the cohort. After ART was initiated, increasing levels of C-reactive protein (CRP), D-dimer, IL-6, IL-7, IL-13, G-CSF, or IL-1RA were associated with increasing hazard of IRIS by time-to-event analysis (each *p*≤0.001). At the time of IRIS onset, multiple proinflammatory cytokine responses were present, including CRP and IL-6. Mortality was predicted by pre-ART increasing IL-17, decreasing GM-CSF, and CRP level >32 mg/l (highest quartile). Pre-ART CRP level >32 mg/l alone was associated with future death (OR = 8.3, 95% CI 2.7–25.6, *p*<0.001).

**Conclusions:**

Pre-ART increases in Th_17_ and Th_2_ responses (e.g., IL-17, IL-4) and lack of proinflammatory cytokine responses (e.g., TNF-α, G-CSF, GM-CSF, VEGF) predispose individuals to subsequent IRIS, perhaps as biomarkers of immune dysfunction and poor initial clearance of CRAG. Although requiring validation, these biomarkers might be an objective tool to stratify the risk of CM-IRIS and death, and could be used clinically to guide when to start ART or use prophylactic interventions.

*Please see later in the article for the Editors' Summary*

## Abstract 2

David Boulware and colleagues investigate clinical features in a prospective cohort with AIDS and recent cryptococcal meningitis after initiation of antiretroviral therapy to identify biomarkers for prediction and diagnosis of CM-IRIS (cryptococcal meninigitis-related immune reconstitution inflammatory syndrome).

## Editors' Summary

**Background:**

Since 1981, AIDS has killed more than 25 million people and about 33 million people are now infected with HIV, which causes AIDS. HIV, which is most often transmitted through unprotected sex with an HIV-infected partner, infects and kills immune system cells. Eventually, the immune system becomes so weak that unusual infections begin to occur. These “opportunistic” infections are infections that take advantage of the opportunity offered by a weakened immune system. One common and deadly opportunistic infection in people affected by AIDS is cryptococcal meningitis (CM), an infection around the brain that is caused by the fungus *Cryptococcus neoformans.* About one million cases of CM occur every year. CM can be treated with a drug called amphotericin but usually recurs unless another drug called fluconazole is taken daily thereafter. HIV therapy is lifesaving by suppressing the HIV virus and allowing immune system recovery. This immune recovery also helps to prevent the recurrence of CM.

**Why Was This Study Done?:**

Unfortunately, HIV therapy can also elicit a serious condition called immune reconstitution inflammatory syndrome (IRIS) in people with CM and AIDS. IRIS is an exaggerated inflammatory immune response that kills up to one-third of affected people. Inflammation, which is characterized by swelling and redness, is the body's first defense against infection, but uncontrolled inflammation causes widespread tissue damage. Experts think that CM-IRIS may be the result of an unbalanced recovery of the immune system leading to an inappropriate immune response to persisting *C. neoformans* fragments and proteins that are slowly cleared from the body over months. Unfortunately, it is impossible to predict which individuals with CM and AIDS will develop IRIS when they are given HIV therapy. In this prospective study, the researchers investigated clinical features and cytokine profiles in a group of Ugandans with AIDS and recent CM for one year after starting HIV therapy to identify biomarkers that could be used to predict and diagnose CM-IRIS. Cytokines are proteins secreted by immune system cells that regulate the immune response; biomarkers are proteins found in the blood that indicate specific diseases.

**What Did the Researchers Do and Find?:**

The researchers enrolled 101 Ugandans with AIDS and recent CM who had not previously received HIV therapy. They compared cytokine patterns in individuals who did and did not subsequently develop IRIS after starting HIV therapy. Overall, 45% of the patients developed IRIS. Deaths occurred in 36% of the patients who developed IRIS and in 21% of those who did not develop IRIS. Patients who developed CM-IRIS after starting HIV therapy had 4-fold higher baseline concentrations of cryptococcal antigen in their blood than patients who did not develop CM-IRIS. Prior to starting HIV therapy, higher levels of the cytokines IL-4 and IL-17 and lower levels of four cytokines—TNF-α, G-CSF, GM-CSF, and VEGF—predicted IRIS development, and an algorithm (formula) based on the baseline levels of seven serum biomarkers was able to group the patients into high, moderate, and low risk of IRIS. After starting HIV therapy, increasing levels of the inflammatory proteins C-reactive protein and D-dimer, and of several cytokines, were associated with an increased risk of IRIS. At the time of IRIS onset, the levels of many proinflammatory cytokine increased. Biomarkers also predicted death after starting HIV therapy with increasing levels of IL-17, decreasing levels of GM-CSF, and a C-reactive protein level of more than 32 mg/l (four times higher than normal) predicted death within one year.

**What Do These Findings Mean?:**

These findings support the hypothesis that some AIDS patients who have a very damaged immune system have a very poor initial immune response and poor clearance of cryptococcus, which predisposes them to IRIS. The findings also identify three distinct phases of IRIS development. Before HIV therapy, a very damaged immune system with a lack of inflammatory responses to infection or inappropriate responses leads to ineffective clearance of the organism and its antigens. After HIV therapy is started, the presence of copious antigens promotes proinflammatory signaling to the immune system. As the immune system recovers proinflammatory immune cells are promoted. Finally, at the time of IRIS, a generalized “cytokine storm” occurs, which is potentially fatal when this inflammation occurs in the brain. The biomarkers identified here as indicators of a predisposition to IRIS need to be validated in more patients in more countries before they can be used as a clinical tool for predicting the risk of IRIS. If they are validated, they could help clinicians decide when to start HIV therapy in patients with AIDS and recent CM, and could guide the use of therapies that could help prevent the abnormal inflammatory responses.

**Additional Information:**

Please access these websites via the online version of this summary at http://dx.doi.org/10.1371/journal.pmed.1000384.

- The US National Institute of Allergy and Infectious Diseases provides information on HIV infection and AIDS
- HIV InSite has information on all aspects of HIV/AIDS, including Knowledge Base Chapters on cryptococcosis and HIV and on the clinical implications of IRIS
- Information is available from Avert, an international AIDS charity on all aspects of HIV/AIDS, including HIV-related opportunistic infections (in English and Spanish)
- The MedlinePlus encyclopedia has a page on cryptococcal meningitis (in English and Spanish)
- AIDS InfoNet provides fact sheets on many HIV/AIDS topics, including a fact sheet on cryptococcal meningitis (in several languages) and treatment guidelines for opportunistic infections

## Introduction

Cryptococcal meningitis (CM), caused by the fungal organism *Cryptococcus neoformans* (commonly termed simply “cryptococcus”) is the most common fatal central nervous system (CNS) infection in persons with AIDS and is the initial AIDS-defining illness in 20%–30% of AIDS patients in Africa, causing 20%–40% of AIDS-attributable mortality [1]–[4]. The US Centers for Disease Control (CDC) estimates approximately 1 million CM cases occur annually, with 70% in sub-Saharan Africa [5]. The incidence of CM among Ugandans with CD4 counts <200 cells/µl is 10% annually without antiretroviral therapy (ART) [1], and without ART, almost all persons with CM die within 6 mo [6]. Although ART availability has led to improved CM survival in Africa [7]–[9], mortality after CM is still high, in part due to paradoxical HIV immune reconstitution inflammatory syndrome (IRIS), an exaggerated inflammatory response causing a subset of persons with recent CM to paradoxically deteriorate on ART in the presence of improving immune function [10]–[14]. Paradoxical CM-IRIS incidence has been reported as 10%–42% among ART-naïve persons with CM, with a pooled average of 18% (95% CI 14%–23%) [7],[14]–[17]. CM-IRIS is of grave concern because of its high incidence and mortality (33%–66%) [7],[14],[17],[18]. In many resource-limited regions, particularly sub-Saharan Africa where CM is the second most common AIDS-defining opportunistic infection [1]–[4], CM-IRIS is a major clinical problem.

The pathophysiology of paradoxical IRIS is largely unknown, but IRIS is hypothesized to be due to dysregulated reconstitution of antigen-specific immunity, leading to an exaggerated immune response to persisting antigens despite microbiologic treatment success [19]–[22]. The antigens, which may be present as intact organisms, dead organisms, or debris [23], evoke pathological inflammatory responses that cause paradoxical clinical deterioration [24]–[26].

**Figure 1:**
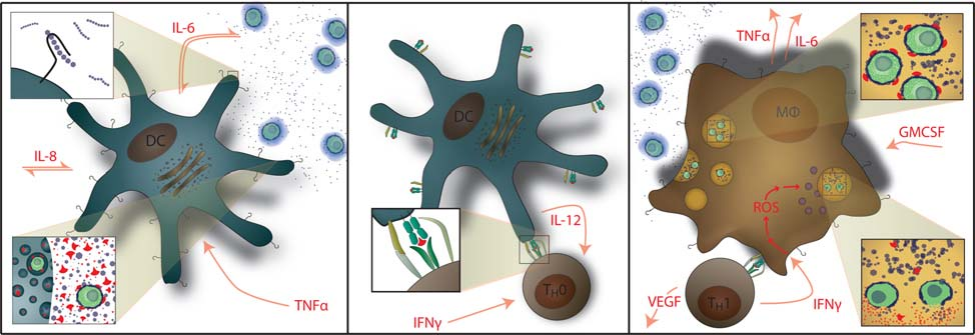
Normal Immunology of Cryptococcosis. **Left:** Myeloid dendritic cells serve as APCs that recognize and phagocytose *C. neoformans* and generate cytokine signals of IL-1β, IL-6, and TNF-α. GXM polysaccharide capsule (i.e. CRAG) activates APCs via Toll-like receptor 4 and CD14. **Center:** Activated dendritic cells migrate to lymphatic tissue and present cryptococcal mannoprotein antigen to naïve T cells via MHC-II receptors. This coupled with CD28 costimulation results in Th1 differentiation. **Right:** Antigen-specific Th1 T cell recognition results in IFN-γ secretion causing classical activation of macrophages with up-regulation of reactive oxygen species (ROS) production; IL-6, TNF-α, IL-8 (CXCL8), CXCL10, fibroblast growth factor (FGF)-2 secretion by macrophages. T cells also secrete additional inflammatory mediators of GM-CSF and VEGF. Th2 T cell responses are not protective. Macrophages also release G-CSF which up-regulates leukotriene synthesis and anticryptococcal activity of neutrophils.

A healthy immune response in non-immunocompromised hosts to cryptococcal infection depends on coordinated interactions between antigen-presenting cells (APCs) and effector T cells, thereby generating an effective type-1 helper T cell (Th1) immune response that clears the infection. Interferon-gamma (IFN-γ) induces classical activation of macrophages to destroy phagocytosed cryptococcus (Figure 1) [27]–[30]. In contrast, Th2 responses are not protective and allow intracellular proliferation of cryptococcus within macrophages, resulting in disseminated infection [31],[32]. Colony-stimulating factors, such as granulocyte colony-stimulating factor (G-CSF) and granulocyte-macrophage colony-stimulating factor (GM-CSF), also up-regulate anti-cryptococcal activity of the innate immune system [33]–[36]. We hypothesize that an ineffectual immune response to cryptococcus occurs in a subset of AIDS patients, and these abnormal responses predispose to the development of IRIS.

We prospectively studied the incidence, clinical presentations, outcomes, and cytokine profiles of CM-IRIS in a cohort of HIV-infected Ugandans initiating ART in order to identify serum biomarkers of inflammation that can be used to predict and diagnose CM-IRIS. Characterizing the abnormal inflammatory responses associated with IRIS will enable the future selection of rational IRIS treatment strategies targeting specific inflammatory pathways.

## Methods

### Participants

One hundred two ART-naïve patients with AIDS and recent CM were enrolled for 12 mo of longitudinal evaluation after ART initiation. One participant was lost to follow up at <2 wk and excluded. Participants with CM were enrolled from May 2006 through September 2009 at Mulago Hospital in Kampala, Uganda [7]. Patients were treated with 0.7–1.0 mg/kg/day of amphotericin for 14 doses, then fluconazole 400 mg daily, and subsequently started on ART as outpatients at the Infectious Disease Institute clinic. ART was initiated at a median of 34 d (interquartile range [IQR] 24–41 d) after CM diagnosis and consisted of: zidovudine, lamivudine, and efavirenz or stavudine, lamivudine, and nevirapine. After ART initiation, participants attended clinic visits biweekly for 3 mo then monthly through 12 mo. Evaluation for IRIS was performed at each scheduled visit and when participants presented acutely. When IRIS was suspected, alternative diagnoses were evaluated by history, examination, laboratory, lumbar puncture, and/or radiological investigations. The International Network for the Study of HIV-associated IRIS (INSHI) consensus working case definition for CM-IRIS was used [37]. Suspected but unconfirmed IRIS events were considered non-IRIS. Each participant provided written informed consent for protocols approved by the institutional review boards at the University of Minnesota, Makerere University, and Uganda National Council for Science and Technology.

### Laboratory Testing

CD4^+^ T cell and plasma HIV RNA measurements were made every 12 wk and at suspected IRIS events. From 2007 onward, CD4^+^ T cells were also measured at 4 and 8 wk of ART. Serum was frozen at −80°C at visits (0, 2, 4, 8, 12, 24 wk and suspected IRIS events). We measured 27 serum cytokines/chemokines in duplicate (Human 27-Plex Panel, Bio-Rad, Hercules, California) according to manufacturer protocol via a Luminex 100 system (Austin, Texas). Plasma D-dimer was measured in 1∶25 dilution by IMUCLONE D-Dimer ELISA (America Diagnostics, Stamford, Connecticut). Serum C-reactive protein (CRP) was measured by reflectance spectrophotometry at the University of Minnesota clinical laboratory. Baseline specimens, collected on the day of ART initiation, were missing from nine patients. Serum cryptococcal antigen (CRAG) titers were batched with each patient's specimens being tested simultaneously in parallel (Immuno-Mycologics, Norman, Oklahoma). At the time of CM-IRIS onset, cerebrospinal fluid (CSF) analysis excluded alternative diagnoses. Investigations included: Gram and acid-fast bacilli (AFB) stains, bacterial and fungal cultures, 16S ribosomal DNA amplification for bacteria [38], and viral culture in Vero cells and RT-PCR amplification for arboviruses performed at the Centers for Disease Control–Fort Collins, Colorado. Patients presenting with pneumonitis were offered bronchoalveolar lavage with separate consent. Histopathology or autopsy specimens always included AFB staining to exclude tuberculosis (TB).

### Statistical Analysis

The primary comparison was between CM patients developing IRIS and control CM patients without IRIS. Non-normally distributed variables (e.g., cytokines, CD4) were tested at baseline by Mann-Whitney *U* and presented as median with IQR. *p*-Values are two-sided with α<0.05 (SPSS 18.0.1, Chicago, IL). Cytokine *p*-values were adjusted for multiple comparisons via Benjamini-Hochberg methods [39]. To determine the risk of future CM-IRIS, pre-ART demographic variables, standard HIV parameters, and log_2_-transformed cytokines were analyzed. A training model was developed in October 2008 after the first 65 participants were enrolled [40], which narrowed the selection from all 29 possible biomarkers to consideration of: CRP, interleukin (IL)-4, IL-8, IL-12, IL-17, G-CSF, GM-CSF, CCL2 (MCP-1), tumor necrosis factor (TNF)-α, and vascular endothelial growth factor (VEGF). To evaluate the additive predictive power of the above cytokine set, we fitted penalized logistic regression models with penalty parameters specified by the Lasso method [41]. Cross-validation was used to estimate the penalty parameters in the model, with 10% of the data removed for each subset estimate. Models were fitted using the “penalized” package in R [42]. The unadjusted models look at the serum biomarkers alone. Six participants were a priori excluded during model development (four suspected IRIS, two TB-IRIS). We also considered an adjusted model including CD4 and the time from CM to ART initiation as unpenalized regression coefficients.

To describe the degree of inflammation occurring at CM-IRIS events, two methods were used. First, cytokine profiles at IRIS events were compared with CM controls time-matched from 4–12 wk of ART (corresponding to the approximate IQR of IRIS timing). Second, we modeled the longitudinal within-patient cytokine changes by Cox regression with a time-dependent covariate to represent each cytokine profile and the profile's evolution over time on ART [43],[44]. The outcome variable in the Cox model was the time to first IRIS event. The “survival package” in R fitted the model on log_2_-transformed data [42]. Sandwich estimates determined the variance of regression coefficients, and the relative IRIS risk was portrayed as the hazard ratio (HR). The *p*-values resulting from each cytokine test were adjusted for multiple comparisons [39]. The Cox model allowed the use of the full longitudinal cytokine profile in order to test for association with the hazard of CM-IRIS events. The HR measures the relative risk of CM-IRIS for each two-fold change in the cytokine expression at any given time point through the first 6 mo of ART.

## Results

### Clinical Features of Cryptococcal IRIS

In this cohort, 57 paradoxical IRIS events occurred in 45 persons (45%; 95% CI 35%–55%; see Figure S1), of whom 35 persons had one event, nine persons had two events, and one person had three events. The median pre-ART CD4 count was 19 (IQR 7–38) cells/µl. Baseline demographic features and both virologic and CD4^+^ T cell responses to ART were similar in patients who did and did not develop cryptococcal IRIS (Table 1). The most frequent clinical manifestation of paradoxical cryptococcal IRIS was aseptic meningitis, which occurred in 30% (30/101) after appropriate clinical responses to initial amphotericin therapy. In these 30 patients, CM-IRIS manifested as headache (87%), photophobia (33%), vomiting (30%), meningismus (27%), papilledema (27%) and seizures (20%). Analysis of CSF showed elevated WBCs in 75% (median 50, range 5–200/µl, lymphocytic predominance), elevated protein in 88% (median 80, range 40–150 mg/dl), and sterile cultures in 96% (27/28, missing two). Each variable was increased compared with pre-ART values (*p*<0.001). Six patients with CM-IRIS presented with seizures that were accompanied by cryptococcoma(s) on CT and/or postmortem exam; three of these patients had concomitant aseptic meningitis. Other paradoxical cryptococcal IRIS events included: pneumonitis with nonproductive cough and radiologic infiltrate (*n* = 14), CNS cryptococcoma without meningitis (*n* = 3), sepsis syndrome with sterile cultures (*n* = 2), and single episodes each of marked lymphadenopathy, retinitis, optic neuritis, and phlyctenular keratoconjunctivitis. CM-IRIS events are summarized and additional clinical details are provided in Text S1. Overall, our results suggest that IRIS occurs in a large percentage of AIDS patients with CM who start ART, and the manifestations of IRIS can be severe, often involving aseptic meningitis and other CNS manifestations.

**Table 1:** Demographic and laboratory parameters in comparison to occurrence of IRIS events.

Parameter	Cryptococcal Meningitis		*p*-Value
	No IRIS	Paradoxical IRIS Event	
*n*	56	45	
BMI	19.4±2.8	20.5±4.6	0.4
Time from CM to ART initiation, d	37±18	36±23	
Median time (IQR)	33 (24–42)	29 (25–41)	0.4
New opportunistic infections	4	3	
Paradoxical IRIS events^a^	—	57	
Death	9^b^	17	0.04
Baseline CD4^+^, cells/µl	34±39	26±26	0.26
Baseline CD8^+^, cells/µl	616±347	589±684	0.8
Baseline CD4:CD8 ratio	0.060±0.059	0.063±0.073	0.16
Baseline eosinophils, eosinophils/µl	307±324	233±270	0.33
Eosinophils, %	13.5%±7.7%	11.3%±6.0%	0.20
Baseline plasma HIV RNA, log copies/ml	5.1±0.6	5.2±0.7	0.9
12 week CD4^+^ T cells/µl	98±77	92±76	0.8
24 week CD4^+^ T cells/µl	101±58	105±75	0.9
48 week CD4^+^ T cells/µl	130±74	175±99	0.056
12 week HIV <400 copies/ml	66%	70%	0.8
24 week <400 copies/ml	88%	90%	0.9
Pre-ART serum CRAG titer,median (IQR) (*n* = 83)	1:128(1:64 to 1∶512)	1∶512(1∶128 to 1∶1024)	0.006

Data are expressed as mean ± standard deviation. *p*-Values are via Mann-Whitney *U* for continuous variables and Fisher's exact test for categorical variables. CRAG is tested by the two independent sample median test.

a Some patients had >1 event, thus the number of events is more than the total listed as the *N* for the group.

b Non-IRIS deaths include: four suspected IRIS events. Two deaths are excluded in persons with virologic suppression with suspected clinical IRIS who refused LPs, with one of these deaths attributed to suicide.

### Association between IRIS and Death

**Figure 2:**
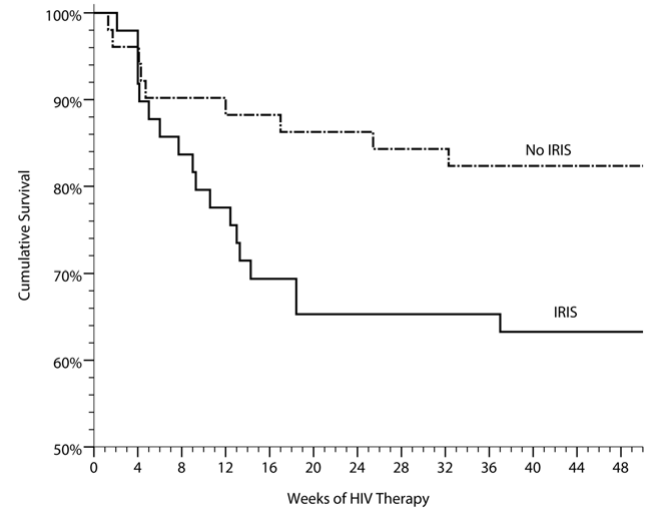
Cumulative survival in persons with prior cryptococcal meningitis newly initiating HIV therapy in Uganda is stratified by the occurrence of cryptococcal IRIS. IRIS was associated with increased mortality (HR = 2.4, 95% CI 1.1–5.3, *p* = 0.035). Included with the controls are three suspected, but unproven cases of CM-IRIS, three unknown causes of death (two suspected pulmonary emboli). Two deaths have been excluded of persons with known virologic suppression with clinical IRIS who refused lumbar punctures to exclude alternative etiologies of their deterioration, of which one of these deaths was attributed to suicide.

In the absence of ART, CM in Africa is associated with high mortality [6],[45]. We analyzed mortality data in our cohort to determine whether the development of IRIS was associated with increased mortality. Overall, 28 of 101 CM patients died (27%) after initiating ART. Mortality was 36% (16/45) in patients with IRIS and 21% (12/56) in those without IRIS (Figure 2). Causes of death were cryptococcal IRIS (*n* = 16), virologic failure and CM relapse (*n* = 1), paradoxical TB-IRIS (*n* = 1), aspiration after AZT-induced severe anemia and lactic acidosis (non-IRIS) (*n* = 1), renal failure (*n* = 1), cerebrovascular event (*n* = 1), suspected but unverified cases of CM-IRIS (*n* = 4), suspected pulmonary emboli (*n* = 2), and one unknown cause of death occurring at home. By time-to-event analysis, mortality was higher among those experiencing cryptococcal IRIS (HR = 2.3, 95% CI 1.1–5.1, *p* = 0.04) than the remainder of the cohort, alone or after adjustment for baseline CD4 (*p* = 0.11 for CD4). Our findings suggest that the development of IRIS is associated with an increased risk for death in patients with recent CM.

### Demographic Risk Factors for IRIS

We sought to determine the risks for IRIS by analyzing demographic and standard HIV parameters. For participants with recent CM, we identified no statistically significant difference among those who did or did not develop IRIS in baseline number or change in CD4^+^ T cell number, in baseline plasma HIV RNA, nor in virologic suppression at 12, 24, 36, or 48 wk (Table1). CD4 recovery is displayed in Figure S2. The lack of differences in these variables pertained to univariate or multivariate analyses, controlling for CRP, body mass index (BMI), cytokine profiles, as well as time-to-event analysis or comparison of quartiles. Thus, the degree of immunosuppression, rate of immune recovery after ART, level of viremia, and degree of viral suppression on ART were not predictors of CM-IRIS in this prospective cohort with very severe immunosuppression.

Median serum CRAG titers when starting ART were 4-fold higher in participants with IRIS (1∶512 cases versus 1∶128 controls, *p* = 0.006). This result is in marked contrast to our previous CSF report from 85 members of this cohort, in whom there were no differences in CSF CRAG titer at time of their initial CM diagnosis between IRIS cases and controls (median CRAG 1∶1024 for both groups) [46]. A pre-ART serum CRAG ≥1∶512 was associated with later CM-IRIS (OR = 4.2, 95% CI 1.7–10.3; *p* = 0.002). Though serum was not available for direct comparison at time of their initial CM diagnosis, the implication is that patients with IRIS likely had poorer antigen clearance in the 5-wk median interval between CM diagnosis and ART initiation.

A concern among many clinicians is that early initiation of ART in patients with CM could lead to an increased incidence of IRIS and increased mortality [47]. We identified no statistical significant difference in the incidence of IRIS in those initiating ART 11–28 d from CM-diagnosis compared with those who waited ≥28 d, (44% versus 55% respectively; *p* = 0.4) nor a statistical difference in survival (*p* = 0.9). Thus, earlier initiation of ART following the diagnosis of CM was not a risk factor for IRIS.

### Predictive IRIS Model

**Figure 3:**
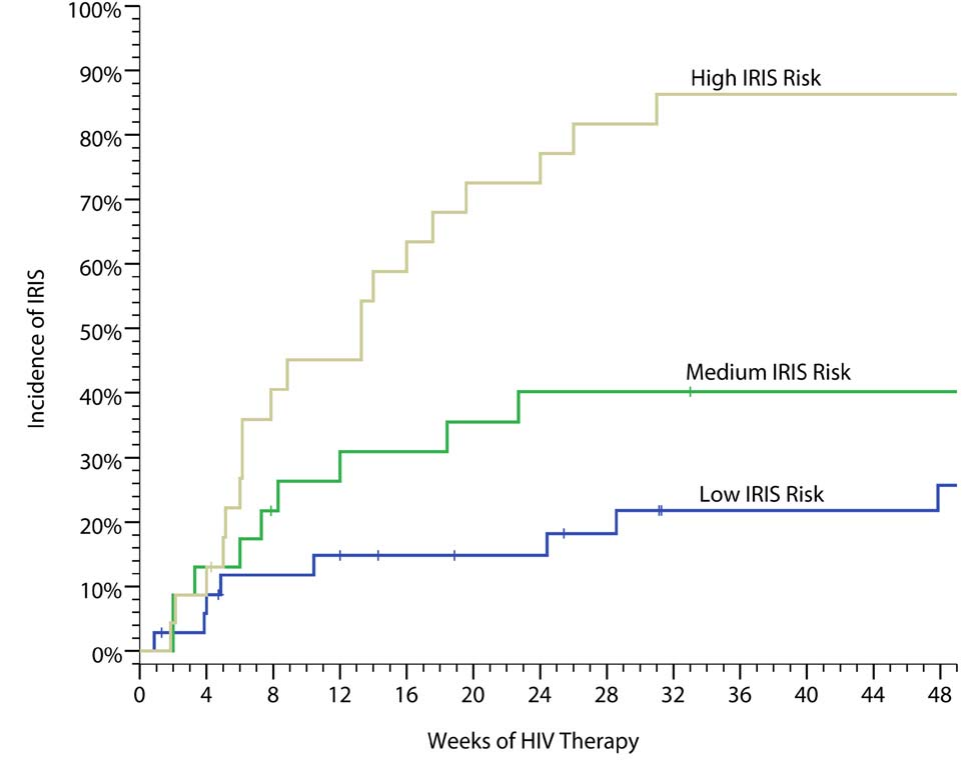
Cumulative incidence of cryptococcal IRIS. The data are based on a weighed predictive biomarker score composed of pre-ART serum levels of seven biomarkers—IL-4, IL-17, G-CSF, GM-CSF, CCL2 (MCP-1), TNF-α, and VEGF—calculated as follows. Probability of IRIS = 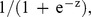 where:
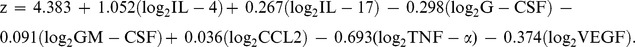
Probability risk is scored from 0 to 1.0 with the categorical classification being defined for low risk (0–0.40), moderate risk (0.41–0.60), and high risk (0.61–1.0). The performance as quantified by the AUC is 0.82 for correct prediction (i.e. C-statistic).

Based on a training set derived from pilot data through 2008 [40], we considered nine possible biomarkers to incorporate into a predictive IRIS model. We used penalized logistic regression with penalties defined by the lasso method to develop a classification model with the best overall fit. Applying this model to our cohort, we were able to stratify persons into high-, moderate-, and low-risks groups with IRIS incidences of 82%, 47%, and 22%, respectively (Figure 3), based on seven serum biomarkers collected on the day of ART initiation (IL-4, IL-17, G-CSF, GM-CSF, CCL2 [MCP-1], TNF-α, and VEGF). The predictive performance (i.e. C-statistic) as quantified by the area under the curve (AUC) of a receiver operating characteristic (ROC) curve is 0.82 for correct prediction (*p*<0.001).

The predictive model had better predictive performance than CRP (AUC = 0.58, *p* = 0.25) or CRAG titer (AUC = 0.67, *p* = 0.004). We considered an adjusted model including the pre-ART CD4 count and the delay from CM to ART initiation. Adjustment did not improve prediction (AUC = 0.83). We also considered the effect on predictive performance by using a more parsimonious model with fewer predictors (Figure S3). The AUC decreased in a linear fashion with inclusion of fewer predictors, such that the AUC was 0.72 for a four-variable model of IL-17, GM-CSF, TNF-α, and VEGF. Although these biomarkers are associated with prediction of IRIS, these factors alone or in aggregate may not be causative of IRIS. However, the roles of these cytokines in immune responses to cryptococcus (Figure 1) suggest that these cytokines may play a role in IRIS pathogenesis. Overall, our results suggest that the risk of IRIS could be stratified on the basis of pre-ART biomarker levels in persons with cryptococcosis.

### Biomarkers Associated with Cryptococcal IRIS

Since predictive models have a different goal and interpretation than do models of association, we also tested for association. We considered the seven biomarkers identified above in a multivariate logistic model to determine their statistical association with IRIS (Table 2). In a multivariate model, future CM-IRIS was associated with increased pre-ART levels of CRP, IL-4, and IL-17 (each p<0.05). Since Th1, rather than Th2 responses, are protective in cryptococcosis [28], a Th2 response to cryptococcus characterized by increased IL-4 production by T cells might be associated with ineffective antigen clearance, leading to a predisposition to the subsequent development of IRIS. Notably, increased pre-ART levels of IL-17, often viewed as a proinflammatory cytokine, were also associated with future IRIS. Moreover, pre-ART levels of the inflammatory marker CRP were increased among patients who subsequently developed IRIS (mean 2.3-fold higher, 95% CI 1.2–3.5, p = 0.03). Persons with CRP >32 mg/l levels (highest quartile) experienced IRIS with an increased incidence of 74% (OR = 3.9, 95% CI 1.3-11.3, p = 0.01) and shorter time-to-event (HR = 3.4, 95% CI 1.8–6.6, p<0.001) compared with persons with CRP<32 mg/l, independent of CD4+ count. Our data suggest that not only is the presence of pre-ART inflammation associated with risk for IRIS, but the type of inflammation is important, with the closest association of IL-4 (Th2) and IL-17 (Th17) responses with a predisposition toward CM-IRIS development.

**Table 2:** Pre-ART serum biomarkers associated with subsequent paradoxical cryptococcal IRIS.

Biomarker(pg/ml)	CM ControlMedian (IQR)	CM-IRIS CaseMedian (IQR)	Odds Ratio per 2-Fold Increase	*p*-Value
TNF-α	73.9 (27.3–130)	31.3 (<1.5–75.0)	0.500	0.001
IL-4	2.8 (2.2–3.5)	3.3 (2.2–4.0)	2.865	0.001
IL-17	66.1 (27.5–125)	47.8 (14.6–131)	1.306	0.018
VEGF	82.7 (47.0–155)	77.3 (34.4–176)	0.688	0.016
G-CSF	25.0 (14.7–41.3)	27.7 (15.0–53.2)	0.742	0.025
GM-CSF	18.8 (4.0–41.4)	8.2 (<1–24.8)	0.913	0.34
CCL2 (MCP-1)	35.7 (9.4–68.8)	24.5 (6.2–84.5)	1.036	0.45

Biomarkers on the day of starting ART that are associated with prediction of subsequent risk of paradoxical CM-IRIS. Risk estimates are calculated by logistic regression using penalty parameters specified by the Lasso method based on log_2_-transformed data [41]. Thus, the odds ratio is reflective of the increased risk per 2-fold increase in the serum biomarker value, adjusting for the influence of each of the biomarkers. *p*-Values are calculated by a multivariate logistic regression.

Although high pre-ART levels of IL-4, IL-17, and CRP were associated with increased risk of IRIS, lower pre-ART levels of VEGF, G-CSF, and TNF-α were also associated with increased risk (*p*<0.05 each). IRIS developed in 79% (11/14) of patients with undetectable serum levels of TNF-α when starting ART, but only 35% (27/78) with detectable TNF-α (OR = 4.9, 95% CI 1.3 to 19.0, *p* = 0.019). TNF-α is involved in innate immunity by activating phagocytosis of cryptococcus by macrophages, antigen processing, and proinflammatory recruitment of additional leukocytes [48],[49], resulting in improved clearance of the organism. Thus, lack of these protective proinflammatory responses at baseline may be associated with subsequent IRIS. In summary, a predisposition to developing IRIS is associated with a paucity of cytokines involved in antigen processing and macrophage function, such as TNF-α, coupled with increased generalized inflammation (e.g., increased CRP, IL-17), lack of antigen recognition by CD4^+^ T cells (e.g., decreased VEGF), and inappropriate Th2 responses (e.g., increased IL-4) before ART.

### Biomarkers on ART

In addition to identifying pre-ART predictors of CM-IRIS, we performed a time-to-event analysis to identify additional predictors of IRIS that evolved in patients while on ART. After ART initiation, analysis of changes in biomarkers during all time points through the first 24 wk of ART showed a significant divergence in inflammatory profiles associated with immune reconstitution between cases and controls that was evident in blood weeks before IRIS events. Increasing levels of CRP, D-dimer, IL-1RA, IL-6, IL-7, IL-13, or G-CSF following initiation of ART were associated with increasing hazard of IRIS (each *p*≤0.001) (Table 3). The pattern of inflammation indicated increasing levels of biomarkers of generalized inflammation such as CRP, IL-6, and D-dimer; inappropriate Th2 responses characterized by elevated IL-13 levels; and elevated levels of growth factors such as G-CSF and the T cell growth and differentiation factor IL-7. The anti-inflammatory mediator IL-1RA increased following ART initiation and before IRIS. The clinical implication of these findings is that these biomarkers could be used to indicate risk for CM-IRIS before the development of IRIS events, providing an opportunity for intervention before clinical deterioration occurs.

**Table 3:** Time-to-event analysis of the hazard of CM-IRIS by serum cytokine profile evolving during ART.

Biomarker	Hazard Ratio per 2-Fold Increase	95% CI	*p*-Value
IL-6	1.591	1.347–1.879	<0.0001
CRP	1.516	1.266–1.816	0.0001
IL-7	1.931	1.408–2.646	0.0004
D-dimer	2.074	1.441–2.986	0.0006
G-CSF	1.442	1.198–1.736	0.0006
IL-1RA	1.416	1.172–1.712	0.0013
IL-13	1.456	1.159–1.830	0.0047
IL-9	1.277	1.050–1.553	0.047
IL-4	1.735	1.065–2.827	0.078
VEGF	1.233	1.011–1.505	0.097
IL-2	1.145	0.970–1.353	0.22
IL-17	1.031	0.911–1.167	0.80
IFN-γ	1.107	0.824–1.486	0.69
TNF-α	1.012	0.876–1.170	0.90

The model incorporated the change from baseline in biomarkers over the first 26 wk of ART as time-dependent variables. *p*-Values are adjusted for multiple comparisons via Benjamini-Hochberg correction [39]. The hazard ratio implies that, as the biomarker increases, the relative risk of having an IRIS event increases. See Table S2 for provision of all biomarkers and unadjusted *p*-values.

### Biomarkers of Inflammation at IRIS Events

**Figure 4:**
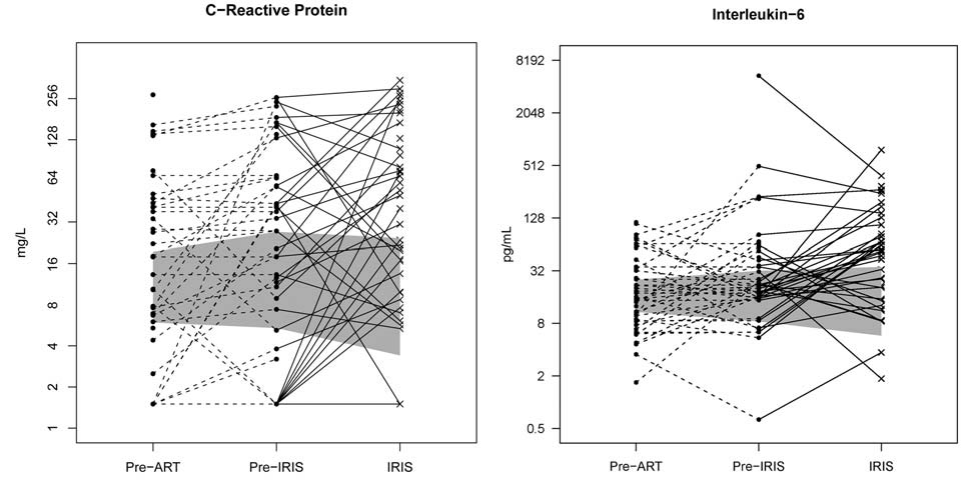
Individual longitudinal increases in CRP and IL-6 from baseline pre-ART, to the time point before IRIS, and at time of IRIS event. The gray shading represents the interquartile range (25^th^ to 75^th^ percentile) of the CM cohort controls without IRIS over the first 8 wk of ART. The median time of IRIS was 8.8 wk. IL-6 and CRP are increased before the IRIS event (*p*<0.001) and at time of IRIS (*p*<0.001).

At time of cryptococcal IRIS, serum biomarkers of inflammation were elevated in almost all IRIS patients over those in time-matched controls (Table 4). However, the pattern of inflammation was heterogeneous with individual variation (Table S1). Early IRIS events were associated with more systemic inflammation than were events occurring after 24 wk. IL-6 was most frequently elevated at time of IRIS events (median 5-fold increase compared with time-matched control participants). CRP levels often correlated with IL-6 levels, as CRP is stimulated by IL-6; and CRP levels were most often increased at IRIS diagnosis compared with levels pre-ART or pre-event (Figure 4; *p*<0.001). In comparing IRIS events with CNS manifestations versus non-CNS IRIS events, there were no statistical differences in any biomarker (each *p*>0.1) with the possible exception of D-dimer being more elevated in non-CNS events (median 3.68 versus 2.22 µg/ml, unadjusted *p* = 0.031). Interestingly, the inflammatory profile distinguished relapse from CM-IRIS in the four cases of culture-positive CM relapse. In the relapse cases, CRP was normal (<8 mg/l), whereas CRP was elevated in 76% of participants with cryptococcal IRIS. These results confirm that IRIS is associated with elevations in markers of inflammation that are large enough to be measured in the serum and can help distinguish patients with CM-IRIS from those with CM relapse before culture results are available.

**Table 4:** Increase in serum biomarkers at the time of first IRIS event compared to time-matched controls.

Biomarker (pg/ml)	Controls Time-Matched Median	IRIS Event (*n* = 43) Median	Geometric Relative Increase^a^	95% CI	*p*-Value
CD4 (cells/µl)	44 (28–82)	77 (30.5–175)			0.077
CRP (mg/l)	8.2 (4.3–18.7)	39.5 (8.4–136)	298%	174%–422%	0.0004
IL-6	9.9 (4.7–22.1)	47.6 (20.7–129.2)	72%	52%–92%	<0.0001
IL-9	30.4 (9.6–51.8)	76.3 (28.7–115)	49%	26%–72%	0.0003
FGF-2	<0.7 (<0.7–1.0)	17.3 (<0.7–72.7)	808%	537%–1080%	0.0004
G-CSF	23.5 (13.9–47.2)	52.2 (26.3–96.6)	19%	9%–29%	0.0005
GM-CSF	3.4 (<1.5–22.7)	21.7 (<1.5–65.5)	111%	61%–160%	0.0009
VEGF	92 (46–170)	203 (108–288)	22%	10%–34%	0.001
D-dimer (µg/ml)	1.6 (1.1–3.1)	3.1 (1.6–4.0)	50%	19%– 81%	0.003
IL-7	12.3 (8.1–20.7)	18.0 (12.8–35.3)	72%	52%–92%	0.003
IL-1RA	109 (50–204)	206 (100–368)	24%	10%–37%	0.005
IL-8	18.2 (9.9–31.9)	32.1 (17.0–62.7)	24%	11%–38%	0.006
IFN-γ	116 (68–175)	167 (103–321)	12%	4%–20%	0.009
IL-17	65.2 (15.1–115)	109.9 (40.6–318)	19%	−7% to 44%	0.018
IL-2	1.2 (<1– 6.8)	4.6 (<1–9.3)	154%	54%–255%	0.042
IL-10	9.5 (4.9–18.1)	13.6 (6.1–32.2)	37%	16%–58%	0.053
TNF-α	41.6 (14.1–103)	73.8 (13.9–111)	12%	−7% to 30%	0.27

The table displays serum cytokines (pg/ml) at time of IRIS event compared to 2:1 cohort time match controls (*n* = 86) from weeks 4 to 12 of ART, corresponding to the approximate interquartile range of the timing of IRIS events. Statistical comparison is via Mann Whitney *U* nonparametric with Benjamini-Hochberg adjustment for multiple comparisons [39], except for CD4, which is not adjusted.

a Relative increase is calculated per the relative risk reduction formula  =  (case – control)/control using the geometric mean of log_2_ transformed cytokines values.

### Biomarkers Predictive of or Associated with Mortality

**Figure 5:**
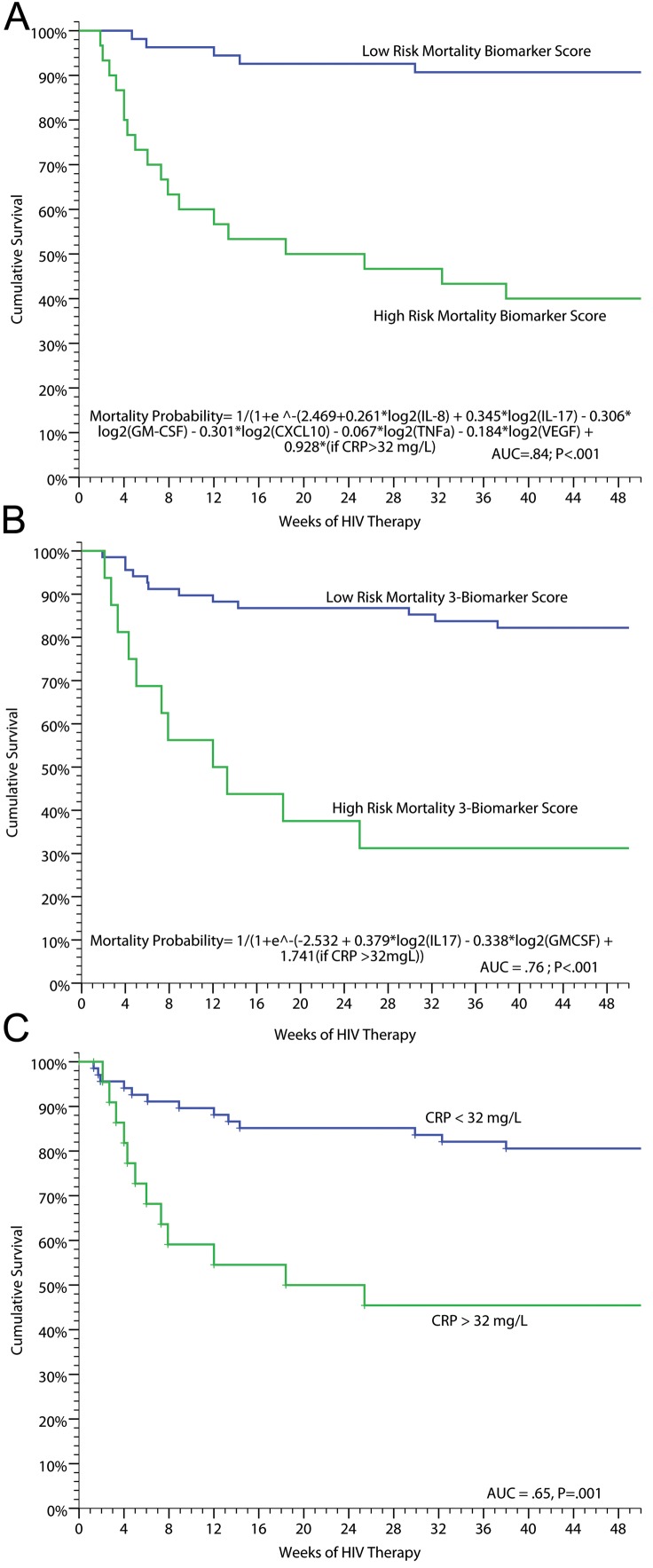
Survival on ART in persons with prior cryptococcosis stratified by their mortality biomarker risk score. (A) Full predictive model (AUC = 0.84). (B) Parsimonious three-biomarker model based on IL-17 (*p* = 0.015), GM-CSF (*p* = 0.022), and categorical CRP >32 mg/l (*p* = 0.004) (AUC = 0.76). With a categorical cutoff point for the model with high risk of probability >0.5 to 1.0, the positive predictive value was 69% and negative predictive value 83% with 48% sensitivity and 92% specificity. (C) Survival stratified by baseline, pre-ART CRP level of<32 or ≥32 mg/l (AUC = 0.65). The three respective ROC curves are presented in Figure S4.

In addition to evaluating predictors of IRIS, we performed analyses to determine if inflammatory profiles could predict death. Using penalized logistic regression with Lasso estimates, we identified baseline pre-ART serum biomarkers that were associated with subsequent mortality on ART. The best fit model consisted of: IL-17, GM-CSF, IL-8, CXCL10, TNF-α, VEGF, and CRP >32 mg/l with an AUC of 0.84 for correct classification with 78% sensitivity, 81% specificity, 60% positive predictive value, and 91% negative predictive value. A more parsimonious three-biomarker model of increasing IL-17 or CRP >32 mg/l, and decreasing GM-CSF, predicted increasing mortality (AUC = 0.76). A weighted score based on these three biomarkers was used to classify high- and low-risk mortality groups (Figure 5). Persons scored as high risk had 69% mortality (i.e., 69% positive predictive value) whereas those with low risk had 83% survival (i.e. 83% negative predictive value), demonstrating the potential clinical utility of the tool to identify patients at increased and decreased risk for death. CRP alone was also clinically useful for predicting risk of death (Figure 5). Participants with a CRP >32 mg/l had 8-fold higher odds of all-cause mortality compared to those with CRP<32 mg/l (OR = 8.3, 95% CI 2.7–25.6, *p*<0.001). Using these biomarkers as clinical tools at the time of ARV initiation could be useful to identify high-risk patients and to monitor them for early therapeutic intervention.

We also performed analyses to determine if levels of biomarkers at time of IRIS events were associated with mortality. Among persons experiencing CM-IRIS, mortality after an IRIS event was associated with increased IL-10 levels (OR = 2.8 per 2-fold change, 95% CI 1.3–5.9, *p* = 0.007) and decreased IL-17 levels (OR = 0.59 per 2-fold change, 95% CI 0.40–0.86, *p* = 0.006) at event compared to persons with IRIS who survived.

## Discussion

In this prospective cohort study of patients with AIDS and recent CM in Uganda, we characterized the incidence and clinical features of CM-IRIS and identified serum biomarkers that can diagnose IRIS and can stratify the risk for IRIS or death. CM-IRIS is a common complication of ART in sub-Saharan Africa, with an incidence reported in the literature ranging from 10% to 31% [11],[14]–[18],[50]. In our cohort with <1% loss to follow up, the incidence of CM-IRIS was 45%, two-thirds of whom experienced characteristic CNS manifestations (30%). Unlike many other forms of IRIS, which produce less dramatic consequences, CM-IRIS is exceptional for its substantial morbidity and mortality [11],[50]–[52]. The 36% CM-IRIS mortality in our study is consistent with other CM-IRIS studies [14],[15],[17],[18],[52],[53]. Such dire outcomes may derive from the increased inflammation that accompanies CM-IRIS in the anatomically constrained compartment of the CNS.

Before this study, it was not possible to predict risk for CM-IRIS. Epidemiologic factors, that were identified in retrospective studies, were not predictive of CM-IRIS in our study, and have not been found consistently across other studies [14],[15],[17],[52]. Similar to a recent ACTG (AIDS Clinical Trial Group) a5164 trial, in our study timing of ART initiation was not associated with IRIS [54]. In contrast, however, we identified specific patterns of biomarkers that were predictive of the development of IRIS and death at initiation of ART, after initiation of ART, and at IRIS event.

Based on our identification of biomarkers that predict CM-IRIS, our theoretical model is one whereby persons predisposed to IRIS have more dysfunctional immune systems that fail to effectively clear antigens from cryptococcus before ART-associated immune restoration. Indeed, persons with IRIS had higher median serum CRAG titers when initiating ART than controls without IRIS (*p* = 0.006), but they had identical 1∶1024 median CSF CRAG titers at their initial CM diagnosis [46]. This result may have implications for customization of timing of ART based on initial induction antifungal therapy prescribed with a longer delay required for less active antifungal therapy (e.g., fluconazole monotherapy).

Further failures of antigen clearance may result from an imbalance in immune regulation, initially characterized by increased production of Th2 cytokines (IL-4, IL-13) and a lack of the proinflammatory mediators (VEGF, TNF-α, G-CSF, GM-CSF) that are required to recruit lymphocytes and activate macrophages and other innate cells to clear *C. neoformans*. Specifically, in neutrophils and macrophages, G-CSF and GM-CSF promote phagocytosis, up-regulation of reactive oxygen species, and intracellular destruction of cryptococcus [33]–[36]. Decreased pre-ART levels of G-CSF, GM-CSF, and TNF-α should result in decreased phagocytosis and promotion of a fertile intracellular environment within macrophages for cryptococcus [36],[49]. Based on our model of CM-IRIS pathogenesis, this inappropriate or ineffective macrophage activation leads to failure to clear cryptococcal organisms and predisposes toward the development of CM-IRIS. In this respect, CM-IRIS pathogenesis may parallel the proposed mechanism of TB-IRIS [55].

A failure of antigen clearance promotes generalized inflammatory signaling to then occur, specifically with IL-6. Compared with time-matched control participants, persons who developed IRIS had elevated levels of CRP before ART initiation, and elevated levels of CRP and IL-6 before and at the time of IRIS events. Our results indicate that the inflammatory signaling response involving IL-6 progressively increased on ART until the development of IRIS. Since IL-6 is the primary stimulant of hepatic CRP production, it is unsurprising that these biomarkers had similar patterns. French and colleagues reported similar increases in plasma IL-6 after a variety of IRIS events or before herpesvirus-related IRIS events, and they verified by flow cytometry experiments that activated macrophages were the primary source of IL-6 [56],[57].

We hypothesize that the abnormal cytokine profiles we observed before CM-IRIS, including elevations of Th2 cytokine (e.g., IL-4, IL-13) and lack of proinflammatory cytokines such as TNF-α, indicate ineffective or inappropriate alternative macrophage activation. In contrast to Th1 responses, which promote IFN-γ–mediated killing of cryptococcus by macrophages [28],[32], IL-4 produced in Th2 cells causes alternative activation of macrophages—typically a response to parasite infections—promoting fibrosis [58]. Alternative activation of macrophages allows for intracellular growth, proliferation, and dissemination of *C. neoformans* [28],[29]. In other fungal processes, IL-4 reverses GM-CSF–mediated macrophage killing of yeast in vitro [59]. In our results, small quantitative increases in IL-4 were associated with large statistical increases in IRIS risk. Since Th1 and Th2 responses are often mutually exclusive, inappropriate increases in Th2 responses (e.g., IL-4, IL-10, IL-13) generate ineffective inflammation and inhibit the protective Th1 responses necessary for efficient antigen clearance. The association of a Th2 bias and risk for IRIS was evident with elevated IL-4 levels at baseline, elevated IL-13 levels on ART before IRIS events, and elevated IL-10 levels at IRIS events as well as an association between increased IL-10 levels at IRIS and subsequent death. The Th2 responses that limit clearance of *C. neoformans* may be driven by preexisting Th2 biases to other concomitant coinfections, such as intestinal helminths or schistosomiasis. This phenomenon might underlie the higher overall incidence of IRIS in our cohort compared to cohorts from temperate regions [14],[15],[17],[50]. Unexplained absolute eosinophilia (>500 cells/µl) was present in over one-quarter of the cohort; however there were not statistical differences in eosinophils or total serum IgE between cases and controls. Overall, our data suggest that Th2 responses to cryptococcus are associated with poor outcomes, including increased risk for IRIS and death.

Risk factors for IRIS extend beyond the Th1:Th2 paradigm. Increased Th17 responses (i.e. IL-17) were a pre-ART risk for both IRIS and mortality on ART. Proinflammatory Th17 cells have been previously hypothesized as important in IRIS pathogenesis [60],[61]. In normal immune homeostasis, a balance between Th17 cells and regulatory T cells (Treg) is crucial. A Th17 imbalance can cause autoimmune diseases. In this balance, IL-6 plays a key role in naïve T cell differentiation inducing Th17 differentiation and inhibiting Treg differentiation in the presence of TGF-β [62]. The copious IL-6 present before IRIS events may alter the balance between Treg and Th17 cells, suppressing Treg differentiation or function during ART-related immune reconstitution.

Additionally, other cytokines that we identified may play a role in IRIS pathogenesis by contributing to ineffective macrophage activation. For example, low pre-ART levels of TNF-α or VEGF were associated with increased IRIS risk. TNF-α, secreted by macrophages and T cells, is particularly important in activating antigen-presenting cells. Absence of TNF-α causes failure of mature dendritic cell activation and recruitment, thereby blunting further recruitment of T cells [63]. Lack of TNF-α could indicate failure to present and/or process antigen and could contribute to ineffective macrophage activation in patients at risk for IRIS. In cryptococcosis, VEGF is secreted by a variety of leukocytes but especially by CD4^+^ T cells during antigen-specific responses to cryptococcal mannoprotein being presented by MHC-II molecules [64]. Decreased VEGF may reflect greater immune dysfunction due to failure of CD4^+^ T cell antigen recognition from antigen-presenting cells. The downstream effects of decreased VEGF would further diminish both chemotaxis and the costimulatory activity of VEGF on IFN-γ–secreting Th1 memory T cells [65]. Thus, decreased VEGF may then diminish the Th1 response and shift the Th1:Th2 balance toward Th2 responses. The decreased levels of TNF-α and VEGF in patients at risk for IRIS may be causes or reflections of impaired macrophage function. Overall, the inappropriate Th2 responses and IL-4 production, coupled with ineffective macrophage activation, suggest that a failure in pathogen recognition and clearance could set the stage for paradoxical IRIS by promoting antigen persistence. Upon eventual immune restoration of more appropriate antigen-specific responses, these responses are exaggerated because of the abundance of uncleared foreign antigen and promotion by IL-6 of a proinflammatory state.

### Study Strengths

Strengths of this study include its prospective design, careful and complete follow-up, and integration of pathophysiologic analyses in the context of characterizing clinical phenotypes. As such, we believe the results reported here are generalizable to cryptococcal IRIS as a pathophysiologic entity. In general, the diagnoses of other forms of IRIS are somewhat subjective, but in CM-IRIS, the identification of IRIS in the CNS is very objective due to the ability to examine the CSF, where the inflammation occurs. A problem arises with non-CNS manifestations of IRIS, which often represent diagnoses of exclusion and depend on the diagnostic capabilities available. In this cohort, we identified several (*n* = 20) non-CNS IRIS events, of which 60% were associated with later CNS-IRIS events or with supportive/definitive histopathology (Text S1). Eight cases (retinitis, optic neuritis, keratoconjunctivitis, pneumonitis; *n* = 5) were probable IRIS diagnoses and unsupported by histopathology or culture (17% of total IRIS cases). Although cryptococcus most often causes meningitis, cryptococcosis is a disseminated disease with systemic CRAG measurable for months in peripheral blood [66]. In our experience here, dead *C. neoformans* causing IRIS reactions were identified in tissue by histopathology of brain, lymphatics, gut, skin, and tongue and were isolated via bronchoalveolar lavage. Thus, non-CNS cryptococcal IRIS events do occur, allowing these “probable events” as likely, but not definitely, attributable to IRIS. In separately considering these non-CNS IRIS events, there were no statistical differences in the inflammatory profiles observed at time of IRIS as compared to CNS-IRIS events, except for possibly D-dimer. In Uganda, there is likely high and ongoing environmental exposure to cryptococcus [1],[4], thus pneumonitis-IRIS could equally be triggered by persisting original antigen or by environmental re-exposure manifesting effectively as an acute hypersensitivity pneumonitis reaction. Pulmonary cryptococcosis often goes unrecognized and/or is suspected as smear-negative TB [67].

### Study Limitations

This prospective observational study has allowed us to characterize the statistical association between cytokine profiles and IRIS. Of course, this does not prove causality; however, the anomalies we report are biologically plausible within the known immunology of responses to cryptococcus. A challenge in pathogenesis studies such as ours is that the inflammatory profiles associated with IRIS are heterogeneous, representing a continuum that is likely dependent on the duration of symptoms, the antigen, and the robustness of the event. In cytokine profiling, six of our participants had minimal immunologic perturbation in peripheral blood even though they clinically deteriorated, presenting with headache and elevated intracranial pressure several months into ART. Thus, some patients fulfilling the IRIS clinical case definition either have localized immune responses that were not detectable in peripheral blood, or they may not have had true immunologic IRIS but instead had delayed complications of CM that were clinically indistinguishable from CM-IRIS. This heterogeneity may have implications for IRIS management and the response to anti-inflammatory therapies. Although this prospective study of CM-IRIS is the largest to date, our biomarker data should be viewed as hypothesis-generating and will require validation in future cohorts. We plan to validate these biomarkers in subjects enrolled in the multi-site Cryptococcal Optimal ART Timing (COAT) trial (NCT 01075152; http://clinicaltrials.gov/ct2/show/NCT01075152) and investigate these biomarkers with regard to timing of ART initiation after CM diagnosis. This validation will give insight on whether altering the timing of ART initiation is a potential intervention to alter high risk or whether other strategies should be pursued. Once validated, creating diagnostics such as multiplex ELISAs or bead-based techniques could move this into clinical use.

### Conclusions

Given the high incidence, morbidity, and mortality associated with CM-IRIS, identifying patients at risk for IRIS may enable interventions to improve management. Three distinct phases of IRIS pathogenesis can be identified (Table 5). First, before ART, a paucity of innate inflammatory responses or inappropriate (Th2) responses promote ineffective antigen clearance. Second, after ART initiation, copious antigen presentation promotes proinflammatory signaling (e.g., IL-6, CRP, IL-7). Third, at the time of IRIS, a generalized cytokine storm occurs. The biomarkers identified here, although requiring validation, may help target interventions to decrease IRIS risk, such as (1) early adjunctive GM-CSF or IFN-γ to increase macrophage function, (2) antiparasitic therapy to eliminate coinfections that promote inappropriate Th2 bias, (3) anti-IL-6 receptor antibody (tocilizumab) therapy to blunt inflammatory signaling, or (4) delaying ART initiation. This study suggests that prediction of IRIS or death may be possible with measurement of pre-ART serum biomarkers.

**Table 5:** Summary of paradoxical cryptococcal-IRIS pathogenesis hypothesis.

Phase	Immunologic Activity	Evidence in CM-IRIS Patients
**Before ART**	• Paucity of appropriate inflammation for cryptococcosis and/or	• ↓ TNF-α, G-CSF, GM-CSF, VEGF in serum↓ IFN-γ, G-CSF, TNF-α, IL-6 in CSF [46]
	• Inappropriate (Th2) responses resulting in:	• ↑IL-4 pre-ART
	• Poor antigen clearance, pre-ART	• Similar CSF CRAG at initial infection [46]Higher CRAG pre-ART
**After starting ART**	• Increasing proinflammatory signaling from APCs due to persisting antigen burden and failure to clear antigen	• ↑ IL6 from macrophages [56] then downstream ↑ CRP production; ↑ IL-7 from APCs
	• Secondary activation of coagulation cascade	• ↑ D-dimer
**At IRIS**	• Cytokine storm of multiple immune pathways of innate and adaptive immune systems	• Th1 ↑ INF-γ, VEGF; TH17 ↑ IL-17Innate: ↑ IL-8, G-CSF, GM-CSF
	• Activation of coagulation cascade	• ↑ D-dimer
	• Neuronal cell activation and damage	• ↑ FGF-2

## Supporting Information

Alternative Language Abstract S1 Translation of the abstract into Spanish by Dr. Jose Debes.
(0.03 MB DOC)

Alternative Language Abstract S2 Translation of the abstract into French by Dr. Anali Conesa Botella.
(0.03 MB DOC)

Alternative Language Abstract S3 Translation of the abstract into Portuguese by DLW and Dr. Jaime Luís Lopes Rocha.
(0.03 MB DOC)

Alternative Language Abstract S4 Translation of the abstract into Russian by Dr. Irina Vlasova-St. Louis.
(0.03 MB DOC)

Alternative Language Abstract S5 Translation of the abstract into Japanese by Dr. Kosuke Yasukawa.
(0.03 MB DOC)

Figure S1 The 45% cumulative incidence of paradoxical CM-IRIS events through 1 y of ART. All patients had prior CM that was diagnosed a median of 5 wk before initiating ART. Censored events are time through non-IRIS deaths (*n* = 5), suspected but unproven IRIS deaths (*n* = 4), unknown causes of death (*n* = 3 of which two were suspected pulmonary emboli), and voluntary ART discontinuation (*n* = 1).
(0.13 MB TIF)

Figure S2 CD4^+^ T cell response in participants with CM-IRIS versus CM controls without IRIS.
Shown are the mean ± SD of the absolute CD4^+^ T cell counts in 101 patients with prior CM, of whom 47 were women and 53 were men with a mean age of 36±8 y. The baseline median CD4^+^ T cell count was 19 (IQR 7–36, range: 1–179) cells/µl increasing by 12 wk of ART to 69 (IQR 44–115) cells/µl, *p*<0.001) with a gradual increase thereafter to a median of 124 (62–175) cells/µl at 48 wk. Plasma HIV RNA of 5.3±0.5 log_10_ copies/ml at baseline achieved suppression (<400 copies/ml) in 70% by 12 wk, 90% at 24 wk, and 87% at 48 wk. There were no statistical significant differences in CD4^+^, CD4 change, or HIV-1 viral load between those who did develop IRIS and those who did not and had uneventful immune reconstitution.
(0.13 MB TIF)

Figure S3ROC Curve for parsimonious IRIS prediction models.ROC curve for more exhaustive or parsimonious models for IRIS prediction using log_2_ transformed biomarkers. AUC ranges from 0.72 to 0.825.IRIS probability  = 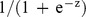, where z is calculated as follows: 
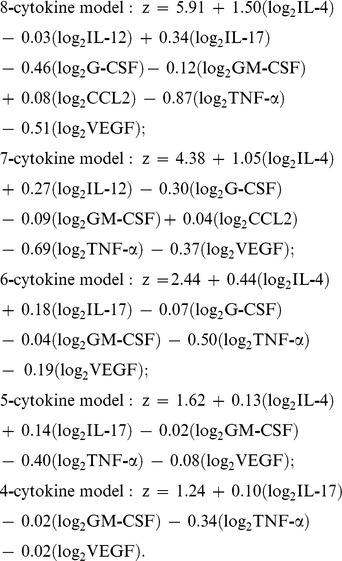

(0.22 MB TIF)

Figure S4 ROC curves for predictive mortality models.
(0.24 MB TIF)

Table S1 Serum cytokine profiles at time of suspected IRIS events.
(0.05 MB PDF)

Table S2 Time-to-event analysis of hazard of IRIS by cytokine profile.
(0.06 MB DOC

Text S1 Clinical spectrum of IRIS events in the Ugandan cohort.
(0.07 MB DOC)

## Abbreviations

AFB: acid-fast bacilli
APC: antigen-presenting cell
ART: antiretroviral therapy
AUC: area under the curve
CM: cryptococcal meningitis
CNS: central nervous system
CRAG: cryptococcal antigen
CRP: C-reactive protein
CSF: cerebrospinal fluid
FGF: fibroblast growth factor
G-CSF: granulocyte colony-stimulating factor
GM-CSF: granulocyte-macrophage colony-stimulating factor
HR: hazard ratio
IL: interleukin
IRIS: immune reconstitution inflammatory syndrome
ROC: receiver operating characteristic
ROS: reactive oxygen species
TB: tuberculosis
TNF: tumor necrosis factor
Treg: regulatory T cells
VEGF: vascular endothelial growth factor
