# Supplementary material for: Clinical Features and Serum Biomarkers in HIV Immune Reconstitution Inflammatory Syndrome after Cryptococcal Meningitis: A Prospective Cohort Study
Source: PLoS Med. 2010 Dec 21;7(12):e1000384. doi: 10.1371/journal.pmed.1000384 (PMC3014618; doi:10.1371/journal.pmed.1000384)
Supplement: Alternative Language Abstract S1 — Translation of the abstract into Spanish by Dr. Jose Debes. (0.03 MB DOC) [file pmed.1000384.s001.doc]

Spanish: Translation of the abstract into Spanish by Dr. Jose Debes.

**Antecedentes**: Aunque la terapia antirretroviral (ART) mejora la supervivencia en personas con meningitis criptocócica (CM) y el SIDA, TAR con frecuencia provoca el síndrome de VIH inflamatorio de reconstitución inmunológica (IRIS), una reacción inflamatoria exagerada y frequentemente mortal que complica la recuperación de la inmunodeficiencia. La patogenia de IRIS es poco conocida, y la predicción de IRIS no es posible.
**Métodos y Resultados**: Analizamos prospectivaente la evolución de 101 pacientes de Uganda sin exposición previa a ART con el SIDA y CM reciente por un año luego de iniciar el ART. Utilizamos ensayos de Luminex múltiplex para comparar los niveles séricos de citoquinas en sujetos que desarrollaron o no IRIS.
 En los pacientes con CM reciente en tratamiento antirretroviral, el IRIS se produjo en el 45%, incluyendo 30% con manifestaciones del sisterma nervioso central (SNC). El tiempo medio de CM-IRIS fue de 8,8 semanas luego de ART. La mortalidad global durante ART fue de 36% con IRIS y de 21% sin IRIS. CM-IRIS mostro estar independientemente asociado a mayor mortalidad(HR = 2.3, IC 95%: 1,1-5,1, p =. 04). Los sujetos que experimentaron IRIS tuvieron cuatro veces más niveles medios de CRAG en suero pre-tratamiento (P =. 006). Los niveles más altos de pre-ART de IL-4 e IL-17, así como el TNF-α menor, G-CSF, GM-CSF, y VEGF predijeron el desarrollo de IRIS en análisis multivariado (ABC = 0,82). Un algoritmo basado en siete biomarcadores en suero pre-TAR es un instrumento importante para la estratificación de alto (83%), moderada (48%), y de bajo riesgo (23%) de IRIS en la muestra. Luego de iniciado ART, el aumento de los niveles de proteína C reactiva (PCR), dímero-D, IL-6, IL-7, IL-13, G-CSF o IL-1ra se asociaron con mayor riesgo de IRIS por análisis de tiempo hasta el evento (cada P <.001). En el momento de IRIS, múltiples reacciones de citoquinas pro-inflamatorias estuvieron presentes, incluida PCR e IL-6. Los predicctores de mortalidad fueron el pre-ART aumento de IL-17, la disminución de GM-CSF, y PCR de nivel> 32 mg / L (el más alto cuartil). El nivel de PCR> 32 mg / L solo mostro asociación con mayor mortalidad a futuro (OR = 8.3, IC 95%: 2.7-25.6, p <.001).
**Conclusiones**: Elevaciones de pre-tratamiento antirretroviral en Th17 y Th2 (por ejemplo, la IL-17, IL-4) y la falta de respuestas de citoquinas pro-inflamatorias (por ejemplo, TNF-α, G-CSF, GM-CSF, VEGF) predisponen a IRIS post-ART, probablemente como biomarcadores de la disfunción inmune y de falta de reacción hacia antígenos criptocócicos. A pesar de que requieren validación, estos biomarcadores pueden ser una herramienta objetiva para estratificar el riesgo de IRIS y de mortalidad que podrían ser utilizados clínicamente para guiar el comienzo de ART o la necesidad de intervenciones profilácticas.
