# Supplementary material for: Clinical Features and Serum Biomarkers in HIV Immune Reconstitution Inflammatory Syndrome after Cryptococcal Meningitis: A Prospective Cohort Study
Source: PLoS Med. 2010 Dec 21;7(12):e1000384. doi: 10.1371/journal.pmed.1000384 (PMC3014618; doi:10.1371/journal.pmed.1000384)
Supplement: Alternative Language Abstract S2 — Translation of the abstract into French by Dr. Anali Conesa Botella. (0.03 MB DOC) [file pmed.1000384.s002.doc]

French: Translation of the abstract into French by Dr. Anali Conesa Botella.

**Contexte**: Bien que la thérapie antirétrovirale (ART) améliore la survie des personnes atteintes de méningite cryptococcique (CM) et le SIDA, ART provoque souvent un syndrome inflammatoire de reconstitution immunitaire (IRIS) contre le VIH, une réaction inflammatoire exagérée et souvent mortelle qui complique le rétablissement de l'immunodéficience. La pathogénèse de IRIS est mal comprise, et il n’est pas encore possible de prédire sa survenue.
**Méthodes et résultats**: Nous avons suivi de manière prospective durant une année suivant l’initiation de ART, 101 individus d’origine Ougandaise, ART naïfs, atteints du SIDA et ayant présenté une CM récemment. Nous avons comparé à l’aide du multiplex Luminex les taux sériques des cytokines chez des sujets qui ont ou n'ont pas développé IRIS.
 Parmi les sujets avec CM récente sous ART, 45% ont développé IRIS, dont 30% avec des manifestations du système nerveux central (SNC). Le délai médian entre le commencement de ART et CM-IRIS était de 8,8 semaines. La mortalité globale sous ART était de 36% et 21% pour les individus ayant développé IRIS ou non IRIS. CM-IRIS était indépendamment associée à la mortalité (HR = 2,3, IC 95%: 1.1 à 5.1, P =. 04). Les sujets ayant développé IRIS sous ART avaient un taux médian de GCRC sérique pré-ART 4 fois plus élevé (P =. 006). La survenue d’IRIS était prédite par une anaylse multivariée par des taux pre-ART de IL-4 et IL-17 élevés ainsi que des taux de TNF-α, G-CSF, GM-CSF, et VEGF abaissés (AUC = 0,82). Un algorithme basé sur sept biomarqueurs sériques pré-ART s’est montré un outil solide pour stratifier le risque de développer IRIS comme élevé (83%), modéré (48%), ou faible (23%) dans la cohorte. Après initiation de ART, l'augmentation des niveaux de CRP, D-dimères, IL-6, IL-7, IL-13, G-CSF ou IL-1ra ont été associés à un risque accru d’IRIS par l'analyse temps-événement (tous p <.001). Au moment d’IRIS, plusieures cytokines pro-inflammatoires étaient présentes, y compris CRP et IL-6. L’augmentation pre-ART de IL-17, la baisse de GM-CSF, une protéine C-réactive (CRP)> 32 mg/L (quartile supérieur) était prédictif de mortalité. Une CRP> 32 mg/L seule a également été associée à un risque accru de décès (OR = 8,3, IC 95%: 2,7 à 25,6, P <.001).
**Conclusions**: Une élévation pré-ART de la réponse Th17 et Th2 (ex.;IL-17 et IL-4) et l’absence de cytokines pro-inflammatoire (ex.; TNF-α, G-CSF, GM-CSF et VEGF) prédisposent au développement d’IRIS, sans doute comme biomarqueurs de dysfonction immunitaire et de faible clairance des antigènes cryptococciques au commencement de ART. Bien que nécessitant d’être validé, ces biomarqueurs peuvent être un outil objectif pour stratifier le risque d'IRIS et de mortalité et pourraient être utilisés en clinique pour guider le moment de commencer ART ou le besoin d'interventions prophylactiques.
