# Supplementary material for: Clinical Features and Serum Biomarkers in HIV Immune Reconstitution Inflammatory Syndrome after Cryptococcal Meningitis: A Prospective Cohort Study
Source: PLoS Med. 2010 Dec 21;7(12):e1000384. doi: 10.1371/journal.pmed.1000384 (PMC3014618; doi:10.1371/journal.pmed.1000384)
Supplement: Alternative Language Abstract S3 — Translation of the abstract into Portuguese by DLW and Dr. Jaime Luís Lopes Rocha. (0.03 MB DOC) [file pmed.1000384.s003.doc]

Português: Translation of the abstract into Portuguese by author Darin Wiesner.

**Fundamento:** Embora o tratamento anti-retroviral (ART) melhore a sobrevida de pessoas com meningite criptocócica (CM) e AIDS, ART frequentemente desencadeia síndrome inflamatória da reconstituição imune (IRIS , uma reação inflamatória exagerada e muitas vezes mortal que dificulta a recuperação da imunodeficiência. A patogênese da IRIS é mal compreendida, e sua previsão não é possível.

**Métodos e Resultados**: Nós acompanhamos prospectivamente 101 indivíduos ART-naive de Uganda com AIDS e CM recentepor um ano após o início da ART e utilizamos ensaios “Luminex multiplex” para comparar os níveis séricos de citocinas em indivíduos que desenvolveram IRIS ou não.
 Em indivíduos com CM recentes sob ART, IRIS ocorreu em 45%, incluindo 30% com manifestações do SNC. O tempo médio para CM-IRIS foi de 8,8 semanas sob ART. A mortalidade total sob ART foi de 36% com a IRIS e 21% sem IRIS. CM-IRIS foi independentemente associada com morte (HR = 2,3, IC 95%: 1,1-5,1, *P* =. 04). Os indivíduos que experimentaram IRIS subseqüente tiveram níveis séricos médios de CRAG pré-ART quatro vezes maior (*P* =. 006). Níveis mais elevadosos no pré-ART de IL-4 e IL-17, assim como níveis mais baixos de TNF-α, G-CSF, GM-CSF e VEGF foram preditores IRIS futura em análise multivariada (AUC = 0,82). Um algoritmo baseado em sete marcadores biológicos pré-ART foi uma ferramenta robusta para estratificar o risco de IRIS na coorte em elevado (83%), moderado (48%) e baixo (23%. Após o início da ART, níveis crescentes de proteína C-reativa (PCR), d-dímero, IL-6, IL-7, IL-13, G-CSF, ou IL-1ra foram associados com risco crescente de IRIS pela análise de tempo para eventos (cada *P* <.001). No momento da IRIS, múltiplas citocinas de respostas pró-inflamatórias estavam presentes, incluindo PCR e IL-6. A mortalidade foi predita por aumento pré-ART da IL-17, diminuição da GM-CSF, e PCR acima de 32 mg / L (maior quartil). Nível isolado de PCR > 32 mg / L foi associado com morte futura (OR = 8.3, 95% CI: 2,7-25,6, *P* <0,001).
**Conclusões**: Elevações pré-ART nas respostasTh17 e Th2 (Ex: IL-17, IL-4) e a falta de resposta nas citocinas pró-inflamatóriass (Ex: TNF-α, G-CSF, GM-CSF, VEGF) predispõem à IRIS subseqüente, talvez como biomarcadores da disfunção imune e pobre depuramento inicial antígenos criptocócicos. Embora haja necessidade de validação, estes biomarcadores podem ser uma ferramenta objetiva para estratificar o risco de IRIS e morte, o que poderia ser utilizado clinicamente para orientar quando começar ART ou a necessidade de intervenções profiláticas.
