# Supplementary material for: Clinical Features and Serum Biomarkers in HIV Immune Reconstitution Inflammatory Syndrome after Cryptococcal Meningitis: A Prospective Cohort Study
Source: PLoS Med. 2010 Dec 21;7(12):e1000384. doi: 10.1371/journal.pmed.1000384 (PMC3014618; doi:10.1371/journal.pmed.1000384)
Supplement: Alternative Language Abstract S4 — Translation of the abstract into Russian by Dr. Irina Vlasova-St. Louis. (0.03 MB DOC) [file pmed.1000384.s004.doc]

Russian: Translation of the abstract into Russian by Dr. Irina Vlasova–St. Louis.

**Справочная информация**: Хотя высокоактивная антиретровирусная терапия (ВААРТ) улучшает выживаемость у больных **СПИДом и** менингитом криптококковой этиологии, ВААРТ часто вызывает осложнение, так называемый воспалительный синдром восстановления иммунитета (ВСВИ). ВСВИ - это парадоксальная реакция иммунной системы, часто со смертельным исходом, которая затрудняет выздоровление пациентов. Патогенез ВСВИ плохо изучен, и прогнозирование ВСВИ в настоящее время не представляется возможным.
**Методы и результаты**: Мы провели перспективное годовое исследование на 101 пациенте со СПИДом, осложненным криптококковым менингитом, в Юганде (Африка). Мы сравнивали уровни цитокинов сыворотки крови у испытуемых (данные Luminex multiplex assays), у которых развился ВСВИ после начала ВААРТ терапии, с пациентами, лечение которых не привело к этому осложнению.
У лиц с криптококковым менингитом в анамнезе (КМ) на фоне лечения ВААРТ, ВСВИ развился в 45% случаев, в том числе у 30% с манифесиацией ЦНС симптомов. Средний промежуток времени до развития ВСВИ составил 8,8 недель лечения. Общая смертность на фоне лечения ВААРТ была 36% - в группе с ВСВИ, и 21% - в неосложненных случаях. Смертность была достоверно повышена у пациентов с ВСВИ и КМ (ОР=2.3; ДИ=1,1-5,1; P =0,04). Перед началом лечения, средний уровень криптококкового антигена был повышен в 4 раза в сыворотках крови у пациентов, впоследствии развивших ВСВИ (P =0,006). По результатам многофакторного анализа, повышенные уровни IL-4 и IL-17, и сниженные уровни TNF-α, G-CSF, GM-CSF, и VEGF явились прогностическими параметрами (AUC= 0,82) в нашем исследовании. Алгоритм, основанный на семи прогностических биомаркерах сыворотки крови, позволил нам рассортировать пациентов в когорты с высоким (83%), умеренным (48%) и низким риском (23%) по развитию ВСВИ. После начала лечения, повышение уровня С-реактивного белка (СРБ), D-димера, IL-6, IL-7, IL-13, G-CSF или IL-1а были ассоциированы с повышенной опасностью развития ВСВИ (P <0,001). Повышение СРБ до уровня > 32 мг/л, так же IL-17, и снижение GM-CSF явились прогностическими маркерами смертельного исхода. Один только уровень СРБ > 32 мг/л коррелировал со смертeльным исходом ВСВИ (OР=8.3; 95% ДИ=2,7-25,6; P <0,001).
**Выводы**: Повышенная активность клеточного иммунного ответа (особенно Tхелпер-17, IL-17, и Tхелпер-2, IL-4) и отсутствие про-воспалительных цитокинов (таких как TNF-α, G-CSF, GM-CSF, VEGF) предрасполагают к развитию ВСВИ, после начала лечения ВААРТ. Их можно считать биомаркерами иммунной дисфункции и персистенции криптококкового антигена. Хотя требуют проверки, эти биомаркеры могут быть объективным инструментом для прогноза ВСВИ, и могут быть использованы в клинике как руководство для начала профилактического лечения.
