# Supplementary material for: Clinical Features and Serum Biomarkers in HIV Immune Reconstitution Inflammatory Syndrome after Cryptococcal Meningitis: A Prospective Cohort Study
Source: PLoS Med. 2010 Dec 21;7(12):e1000384. doi: 10.1371/journal.pmed.1000384 (PMC3014618; doi:10.1371/journal.pmed.1000384)
Supplement: Alternative Language Abstract S5 — Translation of the abstract into Japanese by Dr. Kosuke Yasukawa. (0.03 MB DOC) [file pmed.1000384.s005.doc]

Translation of the abstract into Japanese by Dr. Kosuke Yasukawa.

要旨

**背景**：抗レトロウィルス療法(ART)はクリプトコッカス髄膜炎及びAIDS患者の予後を改善するが、時に免疫の回復過程で過剰な炎症反応を惹起し、致命的となりうる免疫再構築症候群(IRIS)が生じることがある。IRISの発症機序についてはあまり理解されておらず、またどの患者にIRISが生じるのかを予測することは困難である。

**方法と結果**： クリプトコッカス髄膜炎と診断されARTを開始されたウガンダ人AIDS患者101例を、前方視的に1年間追跡し、IRISを発症した群と発症しなかった群の血清サイトカイン値をLuminex Multiplex Assayを用い比較した。

　　ART開始後45%にIRISが発症し、30%が中枢神経症状を生じた。ART開始からクリプトコッカス髄膜炎関連IRISを生じるまでの時間の中央値は8.8週間であった。IRISを生じた群と生じなかった群の死亡率はそれぞれ36%と21%であった。クリプトコッカス髄膜炎関連のIRISは独立して死亡に関連していた(ハザード比2.3、95%信頼区間 1.1-5.1、P値=0.04)。IRISを発症した症例では、ART開始前の血清クリプトコッカス抗原値の中央値は4倍であった(P値=0.006)。多変量解析の結果、ART開始前のIL-4、IL-17の高値及びTNF-α、G-CSF、GM-CSF、VEGFの低値はIRIS発症の予測因子であった(AUC=0.82)。ART開始前の7つの血清バイオマーカーに基づいて作成したアルゴリズムは、ハイリスク(83%)、中等度リスク(48%)、低リスク(23%)とIRIS発症のリスクの層別化に有用であった。時間事象分析によると、ART開始後のCRP、d-dimer、IL-6、IL-7、IL-13、G-CSF、又はIL-1raの上昇は、IRIS発症のハザード比の上昇に関連していた(それぞれP値≦0.001)。IRIS発症時、CRPとIL-6を含む複数の炎症性サイトカイン応答が認められた。ART開始前期間における、IL-17の上昇、GM-CSFの低下、及びCRP値>32mg/L(最高四分位)は死亡の予測因子であり、CRP値>32mg/Lは独立して死亡率と関連していた(オッズ比 8.3、95%信頼区間2.7-25.6、P値<0.001)。

**結論**：ART開始前のTh17及びT2応答の上昇(e.g. IL-17, IL-4)、炎症性サイトカイン(e.g. TNF-α、G-CSF、GM-CSF、VEGF)の低下はIRISの発症の予測因子であり、免疫機能障害とクリプトコッカス抗原が十分排除されていないことを示すバイオマーカーとなっている可能性がある。今後さらなる検証が必要であるが、これらのバイオマーカーはIRIS発症と死亡のリスクを層別化する客観的指標となり得るだけでなく、ARTの開始時期や予防的介入の必要性について考慮するために有用な情報となり得る。
