# Supplementary figures and images for: Clinical Features and Serum Biomarkers in HIV Immune Reconstitution Inflammatory Syndrome after Cryptococcal Meningitis: A Prospective Cohort Study
Source: PLoS Med. 2010 Dec 21;7(12):e1000384. doi: 10.1371/journal.pmed.1000384 (PMC3014618; doi:10.1371/journal.pmed.1000384)

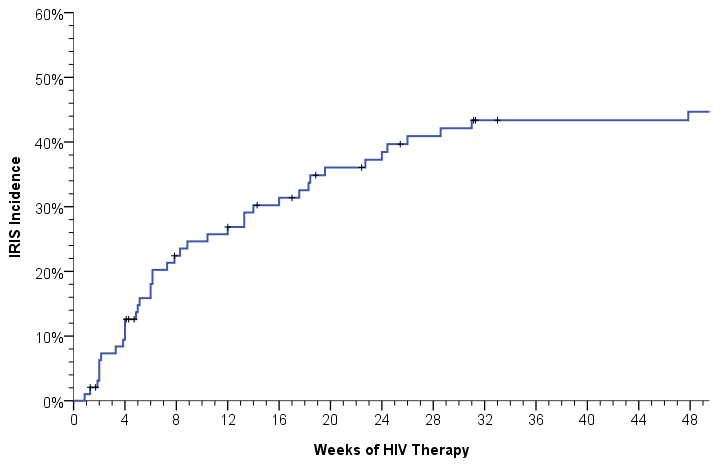

Supplement: Figure S1 — The 45% cumulative incidence of paradoxical CM-IRIS events through 1 y of ART. All patients had prior CM that was diagnosed a median of 5 wk before initiating ART. Censored events are time through non-IRIS deaths (n = 5), suspected but unproven IRIS deaths (n = 4), unknown causes of death (n = 3 of which two were suspected pulmonary emboli), and voluntary ART discontinuation (n = 1). (0.13 MB TIF) [file pmed.1000384.s006.tif]

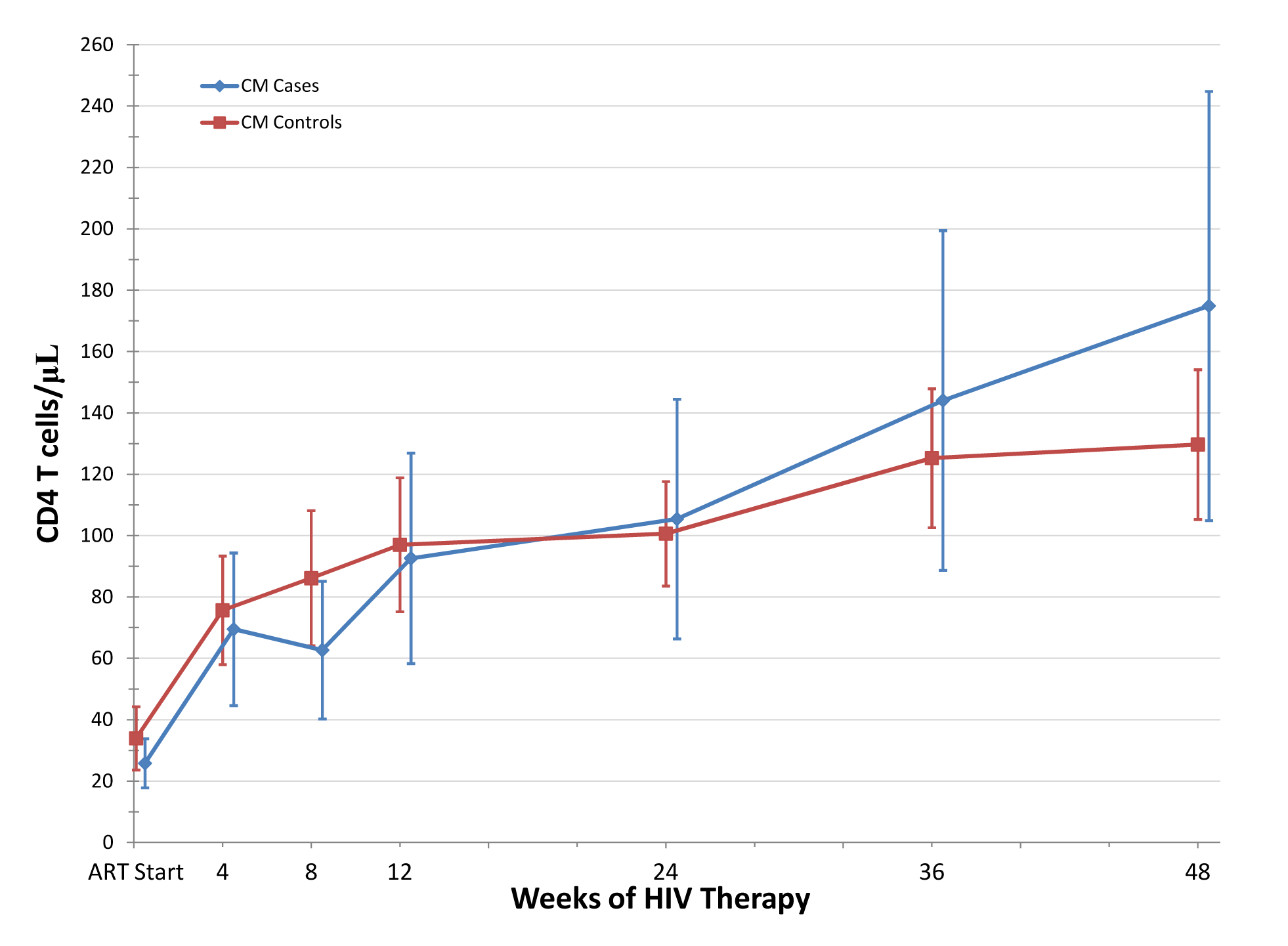

Supplement: Figure S2 — CD4+ T cell response in participants with CM-IRIS versus CM controls without IRIS. Shown are the mean ± SD of the absolute CD4+ T cell counts in 101 patients with prior CM, of whom 47 were women and 53 were men with a mean age of 36±8 y. The baseline median CD4+ T cell count was 19 (IQR 7–36, range: 1–179) cells/µl increasing by 12 wk of ART to 69 (IQR 44–115) cells/µl, p<0.001) with a gradual increase thereafter to a median of 124 (62–175) cells/µl at 48 wk. Plasma HIV RNA of 5.3±0.5 log10 copies/ml at baseline achieved suppression (<400 copies/ml) in 70% by 12 wk, 90% at 24 wk, and 87% at 48 wk. There were no statistical significant differences in CD4+, CD4 change, or HIV-1 viral load between those who did develop IRIS and those who did not and had uneventful immune reconstitution. (0.13 MB TIF) [file pmed.1000384.s007.tif]

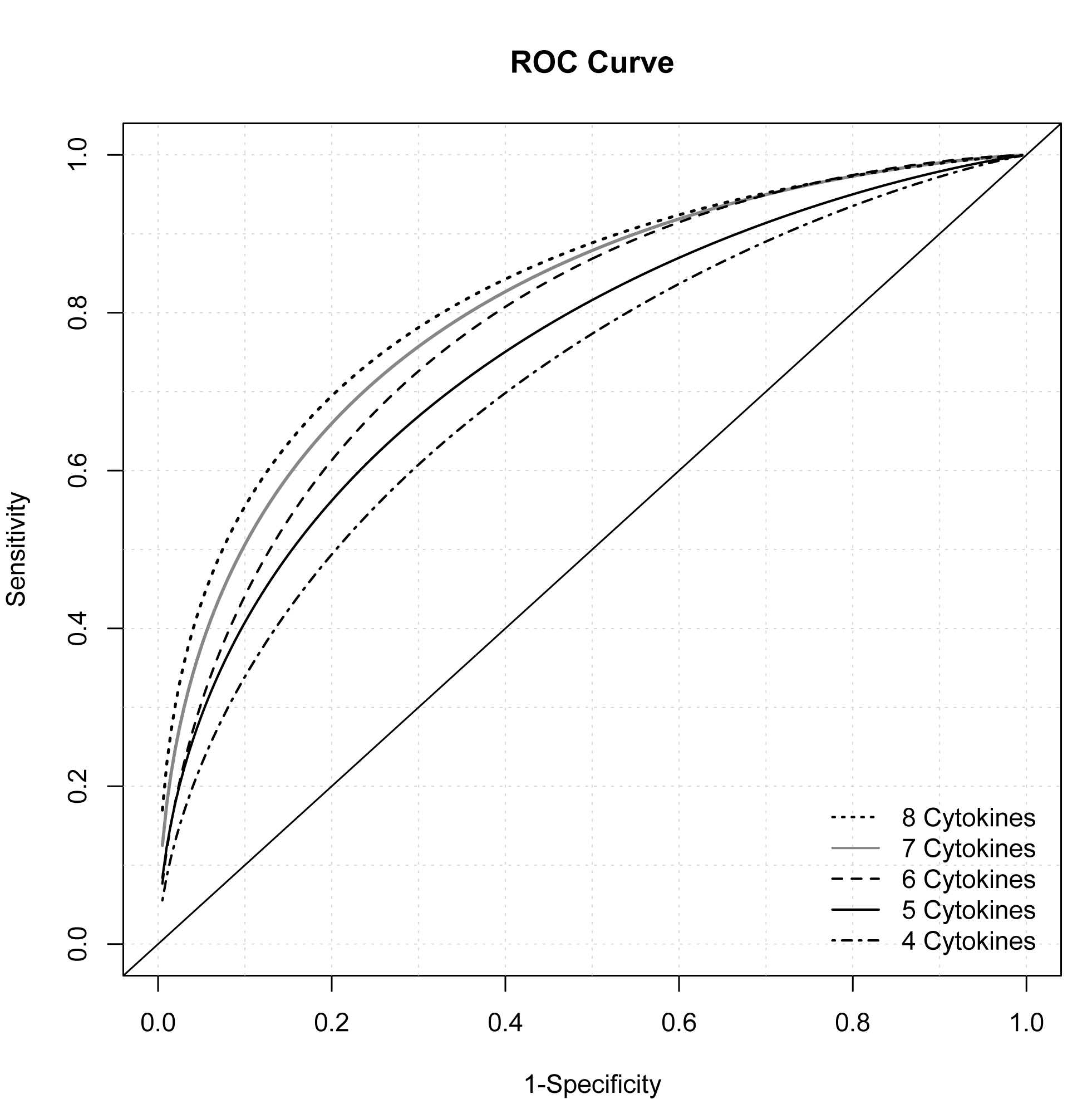

Supplement: Figure S3 — ROC Curve for parsimonious IRIS prediction models. ROC curve for more exhaustive or parsimonious models for IRIS prediction using log2 transformed biomarkers. AUC ranges from 0.72 to 0.825. IRIS probability = , where z is calculated as follows: (0.22 MB TIF) [file pmed.1000384.s008.tif]

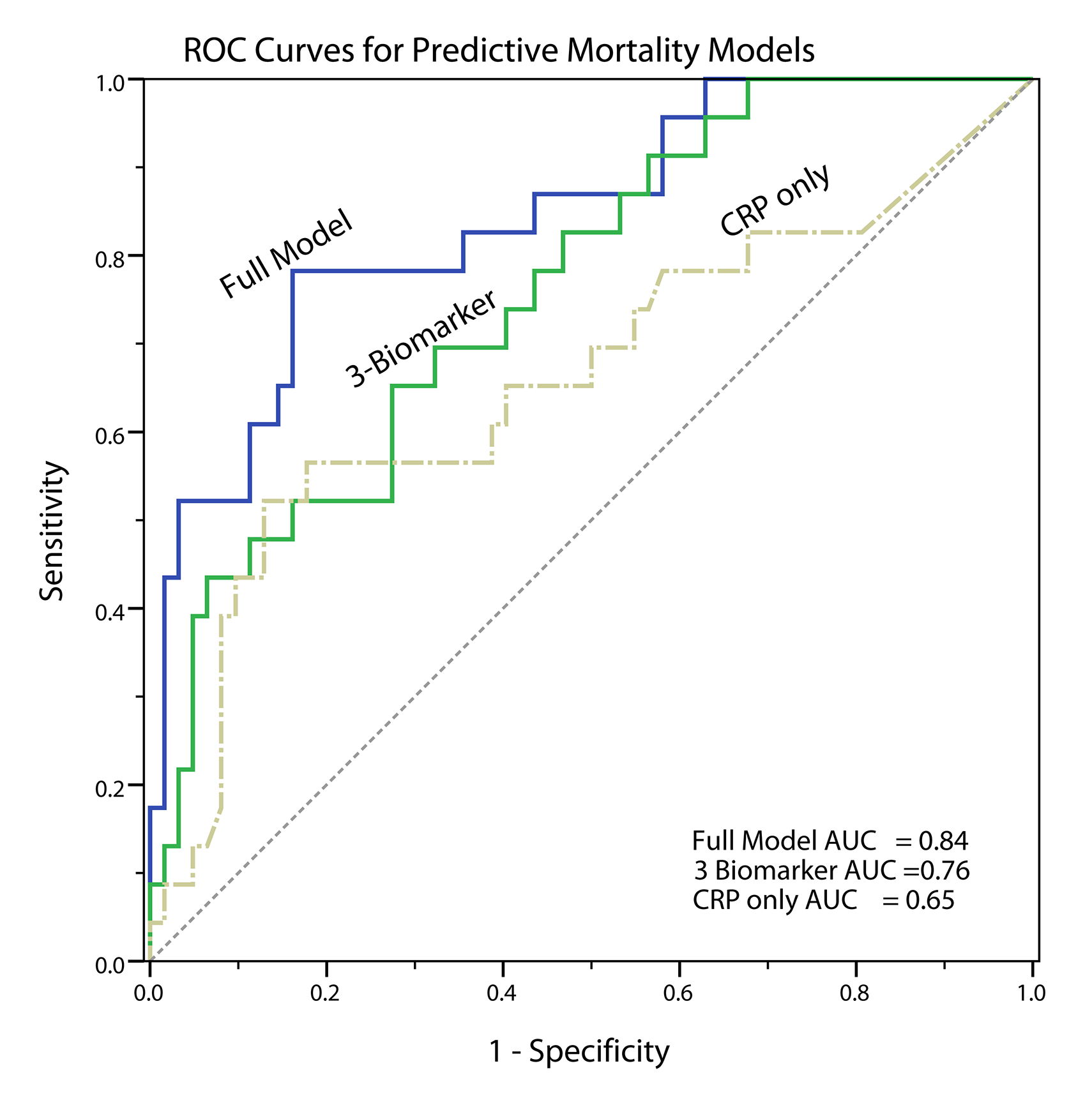

Supplement: Figure S4 — ROC curves for predictive mortality models. (0.24 MB TIF) [file pmed.1000384.s009.tif]
