# Supplementary material for: Clinical Features and Serum Biomarkers in HIV Immune Reconstitution Inflammatory Syndrome after Cryptococcal Meningitis: A Prospective Cohort Study
Source: PLoS Med. 2010 Dec 21;7(12):e1000384. doi: 10.1371/journal.pmed.1000384 (PMC3014618; doi:10.1371/journal.pmed.1000384)
Supplement: Table S1 — Serum cytokine profiles at time of suspected IRIS events. (0.05 MB PDF) [file pmed.1000384.s010.pdf]

Serum Cytokine Profiles at Time of Suspected IRIS Events

Week	IRIS Event	CRP	Ddimer	IL-1b	IL-1ra	IL-2	IL-4	IL-6	IL-7	IL-8	IL-9	IL-10	IL-12	IL-13	IL-15	IL-17	FGFβ	G-CSF	GM-CSF	IFN-γ	CXCL10	MCP-1	MIP-1a	MIP-1b	TNF-α	VEGF	TGF-β
2	Non-CNS	+++	+							+++	+													+			
2	Non-CNS	+++	+++	+		+	+	+++	++		+	++	+++		+	+	++	+		+		+			++	+++	
3.5	Non-CNS	+++	+					+++		++		+++			+			++			+	+++					
5	Non-CNS				+++	+++	++	+++	+++		+++	+	+++	+++				+++									
6	Non-CNS	+++			+++		+++	+++	+++		++			+++			+	+++								+	
6	Non-CNS	+++						+																			N/A
8	Non-CNS	+++	N/A	+++					+++		+		++			+++		+++		+++	++				++		+++
10	Non-CNS	+++	+		+++			+++				+	+								+						
16	Non-CNS	++	++			+++	++			+	+++				+		+++		+++	+++	+			+++		+	N/A
1	CNS-IRIS		N/A																				+				N/A
2	CNS-IRIS	+++		+++				+++	+		++		++		+	+++	++	+++		+		+		+		++	+
2	CNS-IRIS	+++						+																			
2	CNS-IRIS				+	+++	++	+		+	+	+++			+++	++	+++		++	+++		+++	+	+++	+	+++	N/A
3	CNS-IRIS	++	+					+++		+			+								++	++				+	
4	CNS-IRIS	+++	+++		+++			+++		+++		+++						+			+	+++		+			
4	CNS-IRIS												+														+
4	CNS-IRIS	+++						+++				+	+								+	+					
5	CNS-IRIS		N/A												+		+								+		N/A
6	CNS-IRIS	++	++															++				+				+++	
6	CNS-IRIS	++				+++		++				++	++				++			+						+++	++
8	CNS-IRIS	+++	+	+				+++		+	+					+					+						
10	CNS-IRIS	+		+					+							+		+		++					+		+++
12	CNS-IRIS			++							+++					++	+++			+				++	+		N/A
12	CNS-IRIS	+	N/A					+			+	++			+		+								+		N/A
12	CNS-IRIS		N/A																								N/A
13	CNS-IRIS			++		+	+		++		+	+	+++			++		+		++			+++			+	
14	CNS-IRIS				+++			+	+	+	+			+++													
14	CNS-IRIS									+							+++					++		+			N/A
18	CNS-IRIS				+++	+++	+	+	+		+++			+				+									
18	CNS-IRIS	++		++					++		+++		+++			++	+++	+++		+++	++					+++	++
18	CNS-IRIS									+							+++							+			N/A
20	CNS-IRIS	+++	+		+++		+++	++	++					++				++									
24	CNS-IRIS		N/A														++		+			++					N/A
24	CNS-IRIS	+++		+++	+++	+++	+	+++	++			++	+++	+	+++				++	+++		+			+++		
26	CNS-IRIS	+	+					+																			
29	CNS-IRIS				++		+	+++	+++		+++			+++				+					+			+++	
30	CNS-IRIS			+							++					+	+										
30	CNS-IRIS	+										+++			++					+	+	+++		++		+++	N/A
33	CNS-IRIS															+											
36	CNS-IRIS		+														+++		+			+++					N/A
39	CNS-IRIS														+		+++			+		+++					N/A
48	CNS-IRIS	N/A						+		++		++				+	+++			++				+++	+	+++	N/A
15	CM Relapse			++							++		+			++	++	+		+				+++			
25	CM Relapse			+++			+		+		+++				+	+++	+++			+++					+		
2	High ICP					++																+					N/A
5	High ICP		+																								

(+) is >1 SD, (++) is >2 SD, (+++) is >3SD above mean of time matched cohort controls without IRIS
