# Supplementary material for: Clinical Features and Serum Biomarkers in HIV Immune Reconstitution Inflammatory Syndrome after Cryptococcal Meningitis: A Prospective Cohort Study
Source: PLoS Med. 2010 Dec 21;7(12):e1000384. doi: 10.1371/journal.pmed.1000384 (PMC3014618; doi:10.1371/journal.pmed.1000384)
Supplement: Table S2 — Time-to-event analysis of hazard of IRIS by cytokine profile. (0.06 MB DOC [file pmed.1000384.s011.doc]

Table S2

**Time-to-Event Analysis of Hazard of IRIS by Cytokine Profile**

| **Biomarker** | **Hazard Ratio per 2-fold increase** | **95% Conf. Interval** | **Unadjusted P-value** | **Adjusted P-value** |
| --- | --- | --- | --- | --- |
| IL-6 | 1.591 | 1.347 , 1.879 | < 0.0001 | < 0.0001 |
| CRP | 1.516 | 1.266 , 1.816 | < 0.0001 | 0.0001 |
| IL-7 | 1.931 | 1.408 , 2.646 | < 0.0001 | 0.0004 |
| Ddimer | 2.074 | 1.441 , 2.986 | 0.0001 | 0.0006 |
| G-CSF | 1.442 | 1.198 , 1.736 | 0.0001 | 0.0006 |
| IL-1ra | 1.416 | 1.172 , 1.712 | 0.0003 | 0.0013 |
| IL-13 | 1.456 | 1.159 , 1.830 | 0.0013 | 0.0047 |
| IL-9 | 1.277 | 1.050 , 1.553 | 0.0143 | 0.0465 |
| IL-4 | 1.735 | 1.065 , 2.827 | 0.0270 | 0.0780 |
| IL-10 | 1.233 | 1.011 , 1.505 | 0.0390 | 0.0971 |
| VEGF | 1.173 | 1.006 , 1.366 | 0.0411 | 0.0971 |
| IL-2 | 1.144 | 0.981 , 1.333 | 0.0861 | 0.1865 |
| CXCL8 (IL-8) | 1.145 | 0.970 , 1.353 | 0.1098 | 0.2196 |
| IL-12 | 1.249 | 0.938 , 1.662 | 0.1285 | 0.2387 |
| IL-1β | 1.181 | 0.911 , 1.531 | 0.2087 | 0.3617 |
| CCL4 (MIP-1β) | 1.110 | 0.901 , 1.367 | 0.3280 | 0.5329 |
| GM-CSF | 1.037 | 0.939 , 1.146 | 0.4751 | 0.6858 |
| CCL3 (MIP-1α) | 1.049 | 0.917 , 1.201 | 0.4824 | 0.6858 |
| IFN-γ | 1.107 | 0.824 , 1.486 | 0.5011 | 0.6858 |
| IL-17 | 1.031 | 0.911 , 1.167 | 0.6305 | 0.7971 |
| IL-15 | 0.961 | 0.803 , 1.150 | 0.6654 | 0.7971 |
| CXCL10 (IP-10) | 1.048 | 0.841 , 1.306 | 0.6745 | 0.7971 |
| IL-5 | 1.038 | 0.825 , 1.306 | 0.7476 | 0.8451 |
| PDGF-bb | 1.026 | 0.819 , 1.285 | 0.8213 | 0.8897 |
| TNF-α | 1.012 | 0.876 , 1.170 | 0.8679 | 0.9026 |
| CCL2 (MCP-1) | 0.996 | 0.883 , 1.123 | 0.9462 | 0.9462 |

* P-values are statistically significant after adjusted for multiple comparisons via Benjamini-Hochberg FDR correction [39].
