# Supplementary material for: Clinical Features and Serum Biomarkers in HIV Immune Reconstitution Inflammatory Syndrome after Cryptococcal Meningitis: A Prospective Cohort Study
Source: PLoS Med. 2010 Dec 21;7(12):e1000384. doi: 10.1371/journal.pmed.1000384 (PMC3014618; doi:10.1371/journal.pmed.1000384)
Supplement: Text S1 — Clinical spectrum of IRIS events in the Ugandan cohort. (0.07 MB DOC) [file pmed.1000384.s012.doc]

Text S1.

Clinical Spectrum of Immune Reconstitution Inflammatory Syndrome Events in the Ugandan Cohort

| Paradoxical IRIS Event | Timing (weeks) | Adverse Outcome | CRP  (mg/L) |
| --- | --- | --- | --- |
| Aseptic meningitis, cryptococcoma, pneumonitis | 1 | Hemiparesis (resolved) |  |
| Aseptic meningitis^†^, papilledema | 2 |  | 34.9 |
| Aseptic meningitis, OP=320 | 2 |  | 246 |
| Aseptic meningitis, OP=300 | 2 |  | 10 |
| Pneumonitis | 2 |  | 130.9 |
| Pneumonitis, BAL with *C. neoformans* culture | 2 | Recovery | 293.2 |
| Sepsis Syndrome, culture negative | 2.5 | Death in <24 hours | N/A |
| Aseptic meningitis, OP=370 mm | 3 | Death at 8 wks | 75.7 |
| Pneumonitis, ARDS | 3.5 | Resp. failure, Death at day 9 | 301.9 |
| Aseptic meningitis with transverse myelitis | 4 | Rx: anti-TB; Death at 6wks | 110.0 |
| Aseptic meningitis presenting as mania, cryptococcoma on head CT | 4 | Gradual recovery, then Death at 8 wks | 267.3 |
| Aseptic meningitis, cranial nerve VI palsy, OP>550 | 4 | Culture positive meningitis relapse at 15 weeks | 30.3 |
| Pneumonitis; then Aseptic meningitis OP>550 with abdominal lymphadenopathy | 4; 8 |  | 200.9  150.7 |
| Phlyctenular conjunctivitis | 5 |  | 5.7 |
| Aseptic meningitis, CSF culture = 7 CFU/mL with CSF pleocytosis, WBC=5 initial CM =>175 /μL | 5 | Possible Relapse vs. IRIS | 9.8 |
| Cryptococcoma/abscess on post-mortem | 5 | Seizure, Death | N/A |
| Systemic Lymphadenopathy | 6 | Death | 210.0 |
| Retinitis (cryptococcal vs. CMV) | 6 | Death 9 wks later | 232.6 |
| Pleurisy; then aseptic meningitis OP=230 | 6; 18 |  | 69.4 |
| Aseptic meningitis x2 | 6; 24 | Recurrence | 80.6 |
| Pneumonitis, ARDS | 6.5 | Respiratory Failure, Death | 225.3 |
| Pneumonitis of right lower lobe, remote TB history | 7.5 | Spontaneous improvement | N/A |
| Abdominal lymphadenopathy w/ Sepsis, neg AFB | 8 | Death in 3 days | 170.2 |
| Pneumonitis, multi-lobar | 10 | Hospitalization | 348.3 |
| Aseptic meningitis, OP=550 , papilledema | 11 | Prior ↑ICP at 4 weeks |  |
| Aseptic meningitis x2, OP=450 | 10; 14 | Refused initial hospitalization | 40; 33 |
| Aseptic meningitis, OP=320 | 12; 34 | Recurrence, death | 22 |
| Aseptic meningitis, OP=280, seizure | 12 |  | 78 |
| Pneumonitis, altered mental status, TB 8 mo. prior | 12 | Respiratory Failure, Death | N/A |
| Aseptic meningitis OP=240 | 13; 29 | Recurrent IRIS at 29 wks | 21.7 |
| CNS mass lesion (cryptococcoma) | 14 | VZV at 20 wks | 23.6 |
| Cryptococcoma, seizure, lymphadenopathy | 14 |  | 54 |
| Aseptic meningitis x2, OP=380 | 16; 27 | Recurrence, seizure |  |
| Pneumonitis | 16 |  |  |
| Aseptic meningitis | 17 | Prior TB pericarditis at 6 wks | 42 |
| Aseptic meningitis | 18 | Death | 13.5 |
| Aseptic meningitis OP=245; 370 | 18; 30 |  |  |
| Aseptic meningitis with mania, OP=280  New *profound* HPV with rapid onset | 20; 24 | Prior CM Relapse at 12wks | 98.2 |
| Optic Neuritis, blindness, refused LP; then Aseptic meningitis OP=280 | 24; 33 | Recovery of vision w/ steroids | < 3  < 3 |
| Aseptic meningitis, OP=240 | 24 | Psychosis, hallucinations |  |
| Aseptic meningitis OP=360  prior pneumonitis at 24 weeks | 26 | Psychosis at 30wks with normal CSF, uncoordinated speech | 49.9 |
| Aseptic meningitis with bilateral blindness x2 | 28; 38 | Recurrent episode | 19.9 |
| Pneumonitis, normal CXR | 30 | Respiratory failure, Death |  |
| Aseptic meningitis OP=250; then pneumonitis; then recurrent aseptic meningitis OP=230 | 30; 36; 39 | Recurrent episode | 6; 5  7 |
| Aseptic meningitis OP=370 | 48 |  |  |

N/A=not available at time of the IRIS event.

Cryptococcoma(s) were defined as intra-parenchymal enhancing hypodense lesions evident on head CT.

Six cases of suspected IRIS were excluded after diagnostic evaluation. These included 1) CM relapse with positive culture at 2 weeks of ART; 2) elevated intracranial pressure only at 6 weeks 3) CM relapse with virologic failure at 25 weeks; 4) new pulmonary TB at 32 weeks; 5) TB-lymphadenitis at 4 weeks; 6) TB epidural abscess at 16 weeks.

Abbreviations: CRP=c-reactive protein, DD=d-dimer, GM-CSF= granulocyte-monocyte colony stimulating factor, IL=interleukin, IFN=interferon, CXCL10 is also known as IP-10=interferon-gamma induced protein-10, MCP=monocyte chemotaxis protein, MIP= macrophage inflammatory protein, PDGF= platelet-derived growth factor, TNF=tumor necrosis factor; AFB=acid fast bacilli; ARDS=acute respiratory distress syndrome; BAL= bronchoalveolar lavage; CMV=cytomegalovirus HPV=human papilloma virus; TB=tuberculosis; OP=opening pressure (measured in mm H_2_O); wks=weeks

Additional Clinical Details on IRIS Events:

Of the 14 IRIS events manifest as pneumonitis, all subjects had negative sputum smears for AFB and four subsequently developed aseptic meningitis (median 2 weeks). Three subjects with pneumonitis consented for bronchoalveolar lavage to exclude alternative infections; one was *C. neoformans* culture positive at six weeks of anti-fungal therapy and two weeks of ART. Six subjects with pneumonitis had a past history of TB (median 12 weeks of anti-TB therapy, range 9-24 weeks), thus paradoxical TB-IRIS may have occurred, however the timing of pneumonitis in these patients was beyond the 2-4 weeks after ART when TB-IRIS usually occurs [68]. Of the seven remaining pneumonitis events, two rapidly died without alternative diagnosis, and five are best defined as probable cryptococcal-IRIS events without definitive exclusion of alternative etiologies (11% of overall IRIS cases). Five additional subjects had unmasking of ART-associated TB proven by AFB smear (1 pulmonary, 4 extra-pulmonary).

Regarding the suspected but unproven IRIS cases, the first suspected IRIS death was a subject with a headache who gradually became obtunded and comatose at home during the second week of ART. This would be suggestive of increasing intra-cranial pressure (e.g. CM-IRIS vs. CM-relapse), but no clinical assessment was performed before the subject’s death. Two subjects developed a new onset status epilepticus seizure at home and died. The subjects had no prior seizure history. This presentation suggested an inflammatory intra-parenchymal CNS lesion such as a cryptococcoma-related IRIS, but no radiological studies were performed. These suspected but unproven IRIS cases were not counted as IRIS cases in the analysis. A fourth death occurred in an individual with persistent headache and elevated opening pressure (310 mm at 4 weeks of ART) who refused subsequent LPs and hospitalization, dying at home at 6 weeks

In two cases of IRIS-associated pneumonitis, progression of respiratory failure and death occurred despite corticosteroids and empiric anti-bacterial, anti-fungal, and anti-TB therapy. Among the IRIS deaths, half were rapid, within the first 72 hours of hospitalization, while the other deaths occurred 10-21 days after the event. Subjects with severe IRIS received prednisolone 40-60mg daily for 14 days.

The most insightful case was a subject with culture-positive CM-relapse at 12 weeks followed by CM-IRIS at 20 weeks. At time of CM-relapse, CSF revealed 34,000 cryptococcal colony-forming units (CFU)/mL and CSF WBC and protein were unchanged from initial diagnosis (5 WBCs/μL and protein 40mg/dL), no serum cytokine was >1SD different from time-matched control subjects, CRP was 10.8 mg/L, and d-dimer was 1400 ng/mL, also similar to 12-week controls (mean: 1650 +1260 ng/mL). At time of IRIS, however, CRP was 98.2 mg/L, d-dimer was 5650 ng/mL, and multiple pro-inflammatory serum cytokines were elevated >3SD from the mean including IL-1ra, IL6, GM-CSF, and to a lesser degree IL17 and IFN−γ (e.g. >1SD and <3SD elevated). Thus, we observed a clear immunologic difference between CM-relapse and CM-IRIS, with very little inflammation evident at the time of CM relapse, but marked inflammation at the time of IRIS.
